# Denervation impairs regeneration of amputated zebrafish fins

**DOI:** 10.1186/s12861-014-0049-2

**Published:** 2014-12-31

**Authors:** Mariana G Simões, Anabela Bensimon-Brito, Mariana Fonseca, Ana Farinho, Fábio Valério, Sara Sousa, Nuno Afonso, Anoop Kumar, Antonio Jacinto

**Affiliations:** Instituto de Medicina Molecular, Faculdade de Medicina, Universidade de Lisboa, 1649-028 Lisboa, Portugal; CEDOC, Chronic Diseases Research Center, NOVA Medical School, NOVA University of Lisbon, Campo dos Mártires da Pátria, 130, 1169-056 Lisboa, Portugal; Instituto Gulbenkian de Ciência, 2780-156 Oeiras, Portugal; Division of Biosciences, Institute of Structural and Molecular Biology, University College London, London, WC1E 6BT UK

## Abstract

**Background:**

Zebrafish are able to regenerate many of its tissues and organs after damage. In amphibians this process is regulated by nerve fibres present at the site of injury, which have been proposed to release factors into the amputated limbs/fins, promoting and sustaining the proliferation of blastemal cells. Although some candidate factors have been proposed to mediate the nerve dependency of regeneration, the molecular mechanisms involved in this process remain unclear.

**Results:**

We have used zebrafish as a model system to address the role of nerve fibres in fin regeneration. We have developed a protocol for pectoral fin denervation followed by amputation and analysed the regenerative process under this experimental conditions. Upon denervation fins were able to close the wound and form a wound epidermis, but could not establish a functional apical epithelial cap, with a posterior failure of blastema formation and outgrowth, and the accumulation of several defects. The expression patterns of genes known to be key players during fin regeneration were altered upon denervation, suggesting that nerves can contribute to the regulation of the Fgf, Wnt and Shh pathways during zebrafish fin regeneration.

**Conclusions:**

Our results demonstrate that proper innervation of the zebrafish pectoral fin is essential for a successful regenerative process, and establish this organism as a useful model to understand the molecular and cellular mechanisms of nerve dependence, during vertebrate regeneration.

**Electronic supplementary material:**

The online version of this article (doi:10.1186/s12861-014-0049-2) contains supplementary material, which is available to authorized users.

## Background

Zebrafish (*Danio rerio),* a teleost fish with the extraordinary capacity to regenerate several organs and appendages, has been widely used as a model system to study epimorphic regeneration in adults. This organism presents several experimental advantages, such as a rapid and reliable regeneration process and amenability to molecular and genetic manipulation [1,2]. In particular, zebrafish fins are commonly used in regeneration studies. These appendages regenerate promptly after damage through a process that involves the coordination of diverse cellular mechanisms including migration, dedifferentiation, proliferation and patterning, to restore the shape, structure and function of the missing parts [3,4].

Shortly after fin amputation, epithelial cells surrounding the wound migrate to close the stump and establish the wound epidermis (WE) [3]*.* Over time, the WE acquires additional layers by continuous cell migration [2,5,6] to give rise to a specialized epidermis called Apical Epithelial Cap (AEC) [3,7], which is characterized by a robust secretory activity that is especially active in its Basal Epithelial Layer (BEL) [8]. Between 0.5 and 1 dpa, the mesenchymal tissue underneath the AEC loses its organization and cells dedifferentiate and migrate distally towards the amputation plane [3,4]. Subsequently, these cells re-enter the cell cycle to form the blastema, a highly proliferative mass of undifferentiated mesenchymal cells, from which several cell types will differentiate in order to form the missing structures [1,2,9]. Around 2 dpa, some of the blastemal cells lining the epithelial tissue start to differentiate into bone matrix secreting cells (scleroblasts), in a process that is dependent on signals arising from the AEC [6,10-12]. Within the following 10 to 15 days the interactions established between the AEC and the blastema ensure the outgrowth of the regenerating fin and during this period all cell types are re-patterned and a new fin is formed [3]. The BEL has been proposed to play a decisive role in the regulation of growth and patterning throughout regeneration [2,5] due to its proximity to the blastema and to the expression of factors implicated in the signalling to the blastemal cells, such as Wnts (Wingless-type MMTV integration site), Fgfs (Fibroblast Growth Factor) and Shh (Sonic Hedgehog) [10-13].

The establishment and outgrowth of the blastema is a crucial step for tissue repair and its formation is under the control of different factors, including the presence of nerves at the site of injury (reviewed in [14-16]). Nerve dependence of vertebrate appendage regeneration has been studied for many decades. In the amphibian urodeles and in the teleost *Fundulus heteroclitus* it is well documented that upon nerve fibre removal re-innervation of the stump is prevented and consequently limb/fin regeneration is impaired [17-19]. In zebrafish, it was recently demonstrated that the intramuscular injection of Botulinum (a clostridial neurotoxin that inhibits synaptic fusion) alters bone ray outgrowth, patterning, and mineralization after caudal fin amputation [20], reinforcing the idea that nerves are required for proper regeneration process.

Several experiments in amphibians, where limb innervation was removed before or after amputation, have demonstrated that the early events, such as wound healing and initial blastema formation do not require nerve supply, but nerves are essential in promoting and sustaining the proliferation of blastemal cells [21]. These experiments have demonstrated that any component of the nerve (sensorial, motor or sympathetic) has the ability to stimulate regeneration, and have suggested that the signal emanating from nerves should be chemical rather than impulse conduction. Axons have been proposed to release factors into the amputated limbs/fins that promote and sustain the proliferation of blastemal cells [14-16,18] and/or target the WE, which then signals to the underlying mesenchyme, inducing cell dedifferentiation and the establishment of an AEC that is crucial for regeneration [22]. The important relation established between nerve fibres and the WE is well represented by the Accessory Limb Model [23]. This model is based on a set of experiments, which demonstrate that the axolotl limb regeneration is dependent on signals from both the WE and nerve fibres. In this assay, a deviation of a nerve to the site of a lateral wound in the limb, leads to the formation of a blastema-like structure of undifferentiated cells (bump), which does not continue to develop and eventually regresses. However, when a piece of skin is grafted to this wound, at the site of the deviated nerve, an ectopic blastema is formed originating an ectopic limb [23]. Several Fgfs [24-28], members of the Glial Growth Factor (GGF) [29,30], and other candidates, such as Transferrin [31,32] and Substance P [33], have been proposed to act as nerve-derived factors required for amphibian limbs regeneration. In newts, nAG (newt Anterior Gradient) was identified as a secreted molecule able to rescue a denervated blastema and induce regeneration of a denervated limb [34]. A negative model has also been proposed, where the degenerating axons and/or denervated Schwann cells release an inhibitory factor that induces a negative response and inhibits regeneration [35]. Nonetheless, a positive contribution model where nerves release a neurotrophic factor into the regenerating tissue has prevailed in the field [21,36]. Supporting this view is the fact that secretory activity of neurons apparently changes during limb regeneration in the newt, possibly reflecting the formation and transport of trophic substances [37].

Despite the overall knowledge on nerve role in regeneration events, the cellular and molecular mechanisms involved in this process are still not fully understood, in part due to limitations to molecular and genetic manipulation in the model organisms previously studied. In this work we present a protocol for zebrafish pectoral fin denervation followed by amputation that allowed us to address the role of nerve fibres in fin regeneration. Overall, our data shows that in the absence of nerves fins are able to close the wound and form a WE, but cannot establish a functional AEC. Subsequently, fins fail the formation and outgrowth of the blastema, and accumulate several defects. Our study establishes zebrafish as an *in vivo* valuable model to understand the molecular and cellular mechanisms that underlie nerve contribution during vertebrate appendage regeneration.

## Results

### Resection of the brachial plexus is effective to denervate zebrafish pectoral fin

**Figure 1:**
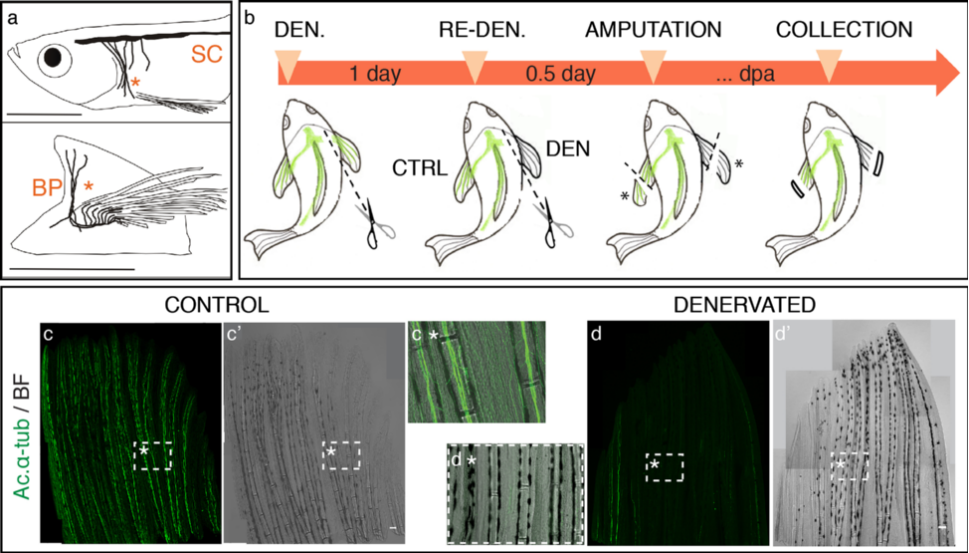
**Adult zebrafish pectoral fin denervation assay. a)** Pectoral fin innervation. The zebrafish pectoral fin is innervated by both sensory and motor nerves that descend from the spinal cord (SC) and enter the pectoral fin region medially, as a combined brachial plexus (BP) (*). Sensory and motor axons then branch to serve the pectoral muscles and fin rays. Sensorial nerves run both along the intra and inter-ray regions (adapted from [38,39]). **b)** Denervation assay. The right pectoral fin was denervated (DEN) by transecting the nerve fibres in the brachial plexus region, while the left fin served as an innervated control (CTRL). In the next day, the right fin was re-denervated to assure total nerve degeneration. After 6–8 hours both fins were amputated and the discarded tissue (*) was collected for ac. α-tub staining. Fish were placed in 33°C water tanks and regeneration was allowed to proceed. Re-denervation took place every day after amputation, to avoid nerve recovery. Regenerates were collected for further analysis at specific time points post-amputation. **c,d)** Pectoral fin denervation efficiency. Staining for ac. α-tub in whole mount fins shows that nerve ablation at the level of the brachial plexus is efficient to deprive pectoral fins from its innervation. An innervated control fin **(c,c’)**, with bundles of axons running in the inter and intra-ray region (c* - magnification of the boxed region in **c)**, contrasts with a denervated fin **(d,d’)**, with fewer or any presence of the axonal marker ac. α-tub, inside and outside bony rays (d*- magnification of the boxed region in **d)**. The images are a projection of confocal optical slices. Scale bar - 100 μm.

To determine whether zebrafish fin regeneration is dependent on a nerve supply, we developed an assay to ablate pectoral fin innervation by surgically removing part of the pectoral fin nerves, in the region of the brachial plexus (Figure1a). The right pectoral fin innervation was surgically removed before amputation, while the left one served as the innervated control (Figure 1b). To evaluate the efficiency of this method, we performed immunofluorescence to label axons, using acetylated α-tubulin (ac.α-tub) as a marker, both in the innervated control and in the denervated discarded fin tissue, collected after amputation (Figure 1c,d). Fins that had been denervated presented very low levels or no ac.α-tub staining, both in the intra and inter-ray tissue (Figure 1d), which confirmed that our denervation protocol is suitable to study nerve dependency during fin regeneration. Due to the difficulty in the identification and access of the brachial plexus, which is deeply inserted in the tissue and runs along blood vessels, the complete elimination of nerves during the procedure was not possible in every fin. Nevertheless, the quantification of our denervation success confirmed that only 4% of the fins had no elimination of nerves, while 14% presented total elimination of nerves and 82% showed only residual presence of nerves. The ac.α-tub staining of the discarded fin tissue collected after amputation allowed us to select the specimens where denervation was efficient to pursue our studies.

### Fin regenerative capacity is affected by denervation

After 1.5 days of fin denervation (a period found to be required to let the axons degenerate) both denervated fins and their contra-lateral controls were amputated and individuals were allowed to regenerate (Figure 1b). Fins were collected and fixed for histological and gene expression analysis at different time points, which according to the literature represent key stages during fin regeneration: 6 hours post amputation (hpa), when the wound healing phase is occurring; 0.5-1 dpa, when the wound healing phase is completed, the AEC is formed, and cells dedifferentiate and migrate to the amputation plane; 1–1.5 dpa, when the blastemal cells start to proliferate; 2 dpa, when the blastemal cells are highly proliferative leading to sustained outgrowth; and 2–5 dpa, when cells are proliferating and differentiating and the new fin is being re-patterned [1,2]. The presence of ac.α-tub, both in the discarded tissue and in the regenerating fins was analysed and the regeneration progress was evaluated. To clarify the effects of nerve absence during regeneration, several parameters were used to characterize control and denervated amputated fins, such as: size and width of rays; apoptosis; morphology; gene expression; and proliferation of blastemal cells.

**Figure 2:**
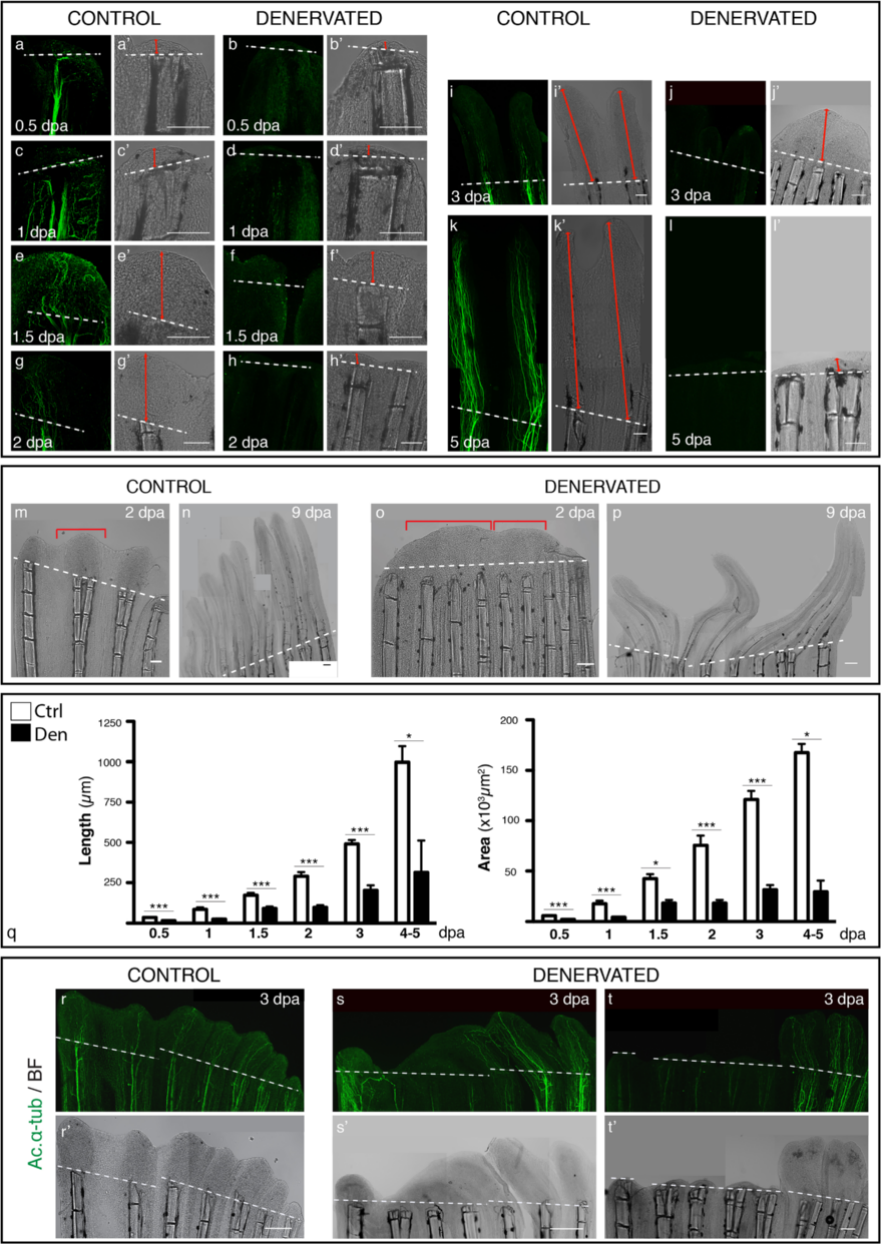
**Analysis of pectoral fin regeneration, upon denervation. a-l)** Staining for ac. α-tub in whole mount fins confirms the absense of nerve fibres at the amputation site of denervated fins. Brightfield images demonstrate the difference in the extent of regeneration among fins **(a’-l’)**. At 0.5 and 1 dpa denervated fins **(b,d)** have a WE that is thinner than controls **(a,c)**. From 1.5 to 5 dpa, denervated fins are not able to form a normal blastema and regenerate **(f,h,j,l)**. In some cases the blastema is completely absent **(h’,l’)**, while in others a smaller and defective blastema is formed and fins regenerate partially **(f’,j’)**. Red arrowed solid lines indicate regenerated tissue length. **m-p)** Defective denervated regenerating fins. Denervated fins regenerate defectively and form “merged blastemas” on adjacent rays. The “wavy” appearance of new control fins, where each ray has its own cone shaped blastema (**m**-bracket), contrasts with denervated fins, where apparently several rays share a single, merged blastema (obracket). At 9 dpa denervated fins with a similar extent of regeneration as the controls **(n)**, present a defective patterning **(p)**. **q)** Quantification of the area and length of regenerated tissue. Measurements taken from the amputation site to the most distal tip reveal a consistent significant reduction (***p < 0.0001, *p < 0.05) of denervated fins in relation to controls. **r-t)** Influence of nerve quantity in fin regeneration. Staining for ac. α-tub. in whole mount fins at 3 dpa show equal innervation and regeneration of control rays within the same fin **(r)**, while denervated fins, the rays with less or no innervation present less or no regeneration **(s,t)**, suggesting that the success of regeneration depends on the quanity of innervation for each ray. **a-q** and **s-t)** The images are a projection of confocal optical slices. Dashed lines mark amputation plane. Scale bar - 100 μm.

Similarly to controls, denervated fins were able to close the wound and form a WE within the first hours after amputation. However, after 0.5 dpa, the WE of denervated fins was thinner than the controls (Figure 2a-d). While in control fins, continuous cell migration led to a thickened epidermis (Additional file 1a,c,e), the WE of denervated fins remained thin, with 2 to 3 layers of epithelial cells (Additional file 1b,d,f). After wound healing, during normal fin regeneration, the mesenchymal cells lying under the WE migrate to the amputation plane to give rise to the blastema (Figure 2e’,g’). However, at 1.5 dpa 28% of denervated fins maintained a thin WE and presented no blastema, while the other 72% formed a smaller blastema and regenerated only partially (Figure 2f’) (Additional file 1 g,h). Similarly, at 2 dpa, in the absence of a proper innervation 45% of denervated fins were not able to regenerate (Figure 2h’), while the other 55% had a small blastema. In later stages, from 3 to 5 dpa, 64% of denervated fins were able to regenerate, although growing less than the controls (Figure 2j’), while the other 36% of denervated fins remained only with a thin WE and with no blastema (Figure 2l’). In occasional cases where denervated fins could regenerate near to almost normal control fin sizes, ray patterning was always defective (Figure 2m-p). In such cases, the fin rays were crooked and thinner (Figure 2p) and consecutive rays often presented “merged blastemas” (Figure 2o). In these malformed blastemas, the usual “wavy” appearance of the new fin, where each ray has its own cone shaped blastema (Figure 2m), was replaced by a continuous tissue spanning several rays (Figure 2o).

**Figure 3:**
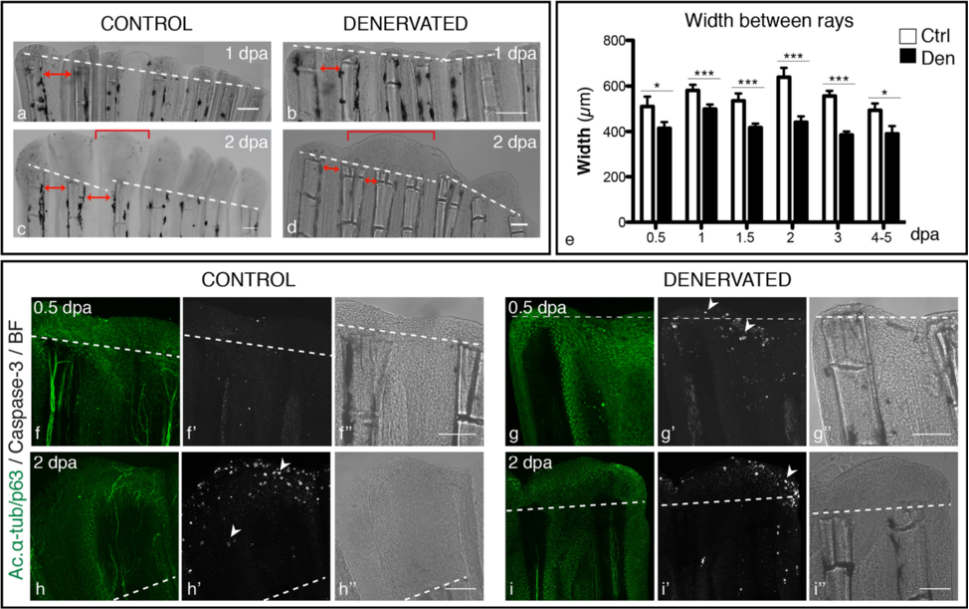
**Width of regenerated fins, upon denervation. a-d)** Brightfield images show that the inter-ray region of denervated fins is reduced in relation to controls. Additionally, “merged blastemas” (bracket) are observed on consecutive rays at 2 dpa **(d)**, in contrast to single-ray blastemas found in control fins **(c)**. Solid red arrowed lines indicate the inter-ray width. **e)** Quantification of inter-ray width. Measurements of rays and inter-ray units show that width of denervated fins is significantly smaller than that of the controls (***p < 0.0001, *p < 0.05). **f-i)** Apoptosis in whole mount amputated fins. Staining for activated caspase3 and ac. α-tub/p63 in whole mount fins activity reveals an increase in apoptotic cells in the WE and in the inter-ray region (arrowhead) of denervated fins, during the first day after amputation **(g’)**. At 2 dpa, activated caspase-3 is expressed in the epidermis of both control **(h’)** and denervated and fins **(i’)**. **a-d**, **f-i)** The images are a projection of confocal optical slices. Dashed lines mark amputation plane. Scale bar - 100 μm.

To quantify the differences between denervated and control fins we measured the area and length of the regenerated tissue, from the amputation site to the most distal tip. The results showed a consistent significant difference between denervated and control fins, with the first ones exhibiting an average reduction of 62% and 53% in area and length, respectively (Figure 2q). In the surgical interventions where the complete elimination of nerves in the fin was not achieved we could observe that rays with less innervation were also the ones presenting less regeneration (Figure 2r-t), suggesting a dependency on the quantity of innervation to fin regeneration efficiency, where the less innervated fins regenerated less tissue. Besides being smaller, denervated fins were narrower than the controls (Figure 3a-d) exhibiting an average reduction of 24% in the distance between pairs of consecutive rays (Figure 3e). Consecutive rays of denervated fins were closer to each other and, as described above, often presented “merged blastemas” (Figure 3d). To investigate whether the narrowing of denervated fins and the thinner WE phenotypes, described above, were due to cell death, we analysed the expression of activated caspase-3 in the WE and inter-ray tissues (Figure 3f-i). We detected more activity of caspase-3 in the WE of denervated fins at 0.5 and 1 dpa, in comparison to control fins (Figure 3f,g) (p = 0.016) where caspase-3 activation was detected only at later stages, during blastema growth (Figure 3h’). We have also observed more caspase in the inter-ray region, but only in the distal tip.

In order to clarify the specific regeneration phases affected by nerve removal, we carried out additional denervation experiments in different time points. When denervation was performed during the formation of the blastema (at 1 dpa) after proper WE establishment, fins presented a smaller regenerate when compared to the control (Additional file 1i). Although significant, the difference between denervated and control fins was not as striking as when fins were denervated before amputation. When denervation was performed after the formation of the blastema (at 2 dpa) we could not detect a significant difference in fin outgrowth between denervated and control fins (Additional file 1j). However, in both cases denervated fins were still narrower than the controls (p < 0.0001 - data not shown) and exhibited some crooked rays. Overall, the results indicate that nerves are crucial to zebrafish fin regeneration, playing a role not only in the initial steps of the regenerative progression, but also exerting their influence during the first two days of regeneration.

### Denervation affects proliferation of mesenchymal cells

During normal fin regeneration, mesenchymal tissue under the AEC loses its organization, dedifferentiate and migrate towards the amputation plane, where it accumulates to form the blastema [3,4]. The presence of Tenascin C, an extracellular matrix protein induced during newt limb and zebrafish fin regeneration upon mesenchyme disorganization [40,41], suggests tissue remodelling in both control and denervated fins (Additional file 2a,b). In addition, morphological analysis of the tissue by Toluidine Blue histology revealed that the mesenchyme under the WE was more disorganized in comparison to more proximal tissue (Additional file 2c,d), with cells presenting a more elongated shape, suggesting cell migration. These results indicate that the initial tissue remodelling can still occur upon amputation of denervated fins, but the subsequent blastema formation and growth is disrupted.

**Figure 4:**
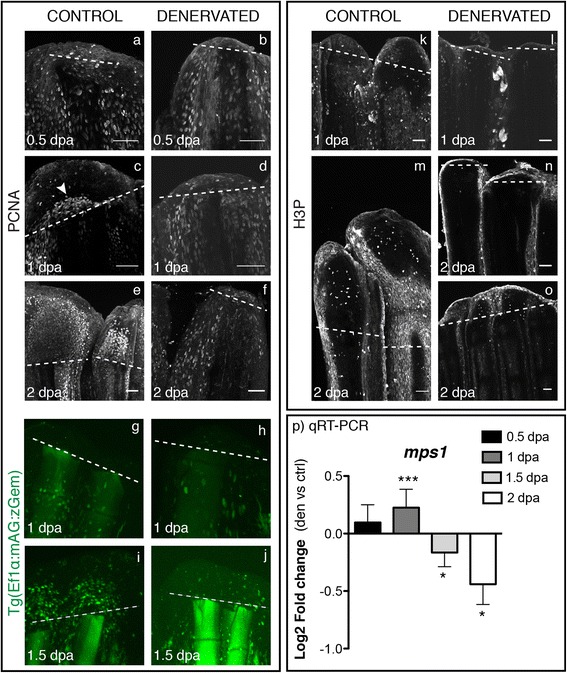
**Analysis of cell cycle markers in mesenchymal cells, upon denervation. a-f)** Staining for PCNA in whole mount fins shows equal expression in epidermal and mesenchymal cells of both control and denervated fins at 0.5 dpa **(a,b)**. At 1 dpa, PCNA-positive cells start to accumulate at the level of amputation in the region that will give rise to the blastema in control fins (**c** - arrowhead), which is not observed in denervated fins **(d)**. At 2 dpa control fins show an accumulation of PCNA-positive cells in the blastema region **(e)**, while denervated fins resemble as 0.5 dpa fins **(f)**. **g-j)** Live imaging with the Tg(Ef1α:mAG:zGem). At 1 dpa Geminin-positive cells are equally expressed in control **(g)** and denervated fins **(h)**. GFP nuclei started to be evident in the mesenchyme above the amputation plane of control fins at 1.5 dpa **(i)**, but not in the denervated ones **(j)**. **k-o)** Staining for H3P in whole mount fins. H3P starts to be expressed at 1 dpa in some mesenchymal cells of control fins **(k)**. At 2 dpa H3P-positive cells are present only in control fins **(m)** and in reduced number in partially regenerating denervated fins **(o)**. Note: H3P has a non-specific label in distal epidermal cells, as previously reported [42]. **a-o)** The images are a projection of confocal optical slices. Dashed lines mark amputation plane. Scale bar - 50 μm. **p)** qRT-PCR for*mps1. Mps1* levels of expression increase in denervated fins at 1 dpa and decrease at 1.5 and 2 dpa, in relation to controls (***p < 0.0001, *p < 0.05).

In order to determine how the cell cycle was affected upon fin denervation, we analysed the expression of three different cell cycle progression markers (PCNA-Proliferating cell nuclear antigen, Geminin and H3P-Phosphorylated histone H3) and a checkpoint regulator (*mps1/ttk*), which is specifically expressed in the highly proliferative fin blastema cells [42]. PCNA, which is expressed throughout G1 to M phases enabling the detection of a wide range of proliferating stages [43], was equally expressed in the epidermal and mesenchymal cells of both control and denervated fins, at 0.5 dpa (Figure 4a,b). In control fins, at 1 and 2 dpa, PCNA staining accumulated in the mesenchyme above the level of amputation, in the region that gives rise to the blastema (Figure 4c,e), while in denervated fins, PCNA expression at later stages maintained the same pattern as 0.5 dpa (Figure 4d,f). Analysis of Geminin, which accumulates in the nuclei through S/G2/M phases of the cell cycle, was possible by using a transgenic zebrafish [Tg(Ef1α:mAG:zGem)] that labels cell nuclei GFP during S/G2/M phases [44]. At early time points, no differences in Geminin expression were observed in the mesenchyme of denervated fins in relation to controls (Figure 4g,h). However, after 1.5 dpa, Geminin expression in the nuclei of denervated fins was reduced (Figure 4j) and an accumulation of Geminin-positive cells was observed above the amputation plane of control fins (Figure 4i). Mitotic cells, positive for H3P, were first detected at 1 dpa in some mesenchymal cells (Figure 4k) and later maintained in the blastema and intra-ray mesenchyme of control fins (Figure 4m). However, few positive cells were observed in denervated fins, and a significant difference exists between the number of positive H3P cells between control and denervated fins at 1.5 and 2dpa (Figure 4n,o) (p = 0.0013). In addition, the levels of expression of *mps1,* a kinase that regulates the mitotic spindle assembly checkpoint (G2/M Transition) in eukaryotic cells [42,45] and that ensures genomic integrity by delaying cell cycle progression in response to a range of stress agents [46], were significantly increased in denervated fins at 1 dpa, followed by a decrease at 1.5 and 2 dpa (Figure 4p).

Together, these results indicate that upon amputation cell cycle re-entry of the mesenchyme is not affected by denervation. As the cell cycle markers PCNA and Geminin exhibit similar mesenchyme expression, both in denervated fins and in controls, it is likely that the G1-S and S-G2 transitions were not impaired by the lack of innervation. However, the transition G2-M seems to be affected because the expression of H3P, which marks only cells in the M phase, was reduced in denervated fins. The increase of *mps1* levels of expression at 0.5 and 1 dpa on denervated fins is also compatible with a G2/M arrest.

### Structure and signalling of the AEC is altered on denervated fins

In order to better understand the WE phenotype, as well as to assess the formation of a functional AEC, we analysed the expression of several epidermal regulators in control and denervated fins.

**Figure 5:**
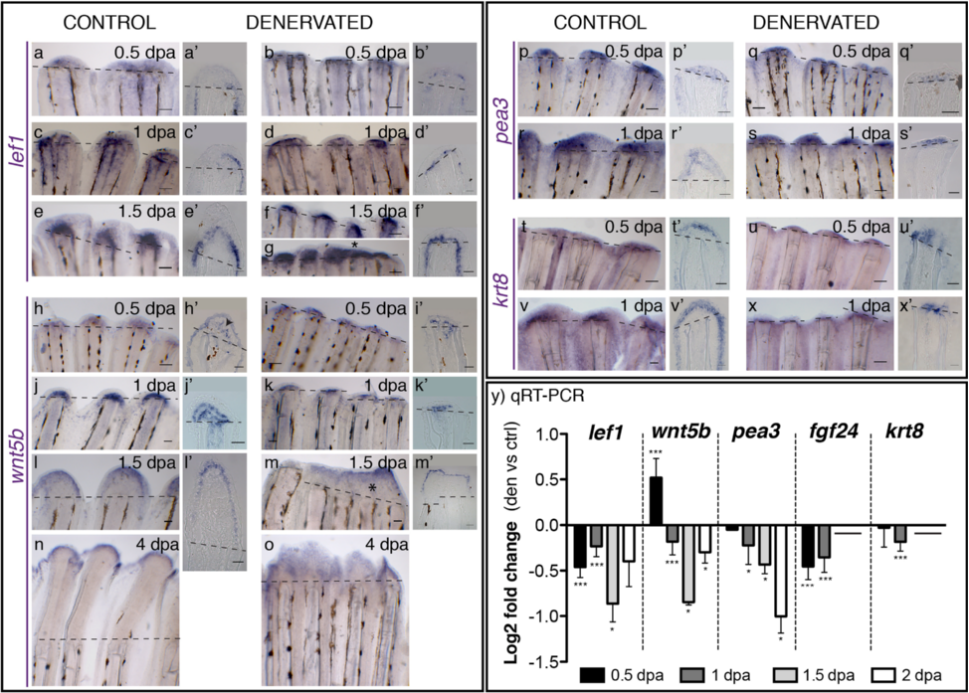
**Gene expression in the WE of control and denervated fins. a-x)** mRNA ISH on whole mount amputated fins: *lef1, wnt5b, pea3, krt8*. **a’-x’)** Longitudinal sections of the rays using whole mount ISH. **a-g)** *Lef1* is expressed from 0.5 to 1.5 dpa in the BEL of both control **(a,c,e)** and denervated fins **(b,d,f,g)**. At 1.5 dpa *lef1* is expressed in the inter-ray region of non-regenerating **(f)** and partially regenerating denervated fins, where it forms what seems a shared BEL on contiguous rays (g*). **h-o)** *Wnt5b* is expressed in the WE of both control **(h,j,l,n)** and denervated fins **(i,k,m,o)** from 0.5 to 4 dpa. [The arrowhead in h’ indicates expression, staining in the WE is an artefact]. In denervated fins, after 1.5 dpa, *wnt5b* presents a spread and de-regulated expression domain (m). **p-s)** *Pea3* is expressed in both control and denervated fins at 0.5 and 1 dpa, with a reduced expression in denervated fins **(q,s)**. In controls pea3 is expressed in the whole WE **(p’,r’)**, while in denervates is restricted to the distal WE cells **(q’,s’)**. **t-x)** *Krt8* is expressed in both control **(t,v)** and denervated fins **(u,x)** at 0.5 and 1 dpa, with a reduced expression in denervates **(u,x)**. While in controls *krt8* is strongly expressed in the whole WE **(t’,v’)**, in denervates it is restricted to some epidermal cells in the distal tip **(u’,x’)**. **a,a’-x,x’)** Dashed lines mark amputation plane. Scale bar - 100 μm in whole mount; 25 μm in sections. **y)** qRT-PCR for genes expressed in the WE. qRT-PCR analysis shows a decrease in the expression levels of analysed genes on denervated fins, in relation to controls, from 0.5 to 2 dpa, except for *wnt5b*, which is increased at 0.5 dpa. Note: ***p < 0.0001, *p < 0.05. Data not evaluated for *krt8* and *fgf24*, at 1.5 and 2 dpa.

We have first investigated the presence of Lef1*,* a transcriptional target of the canonical Wnt signalling pathway, known to be up regulated early in the WE and maintained in the BEL during the formation and outgrowth of the caudal fin blastema in more proximal cells [5,11]. Lef1 was expressed in the BEL of both control and denervated fins (Figure 5a-g and Additional file 3) with different domains of expression. At 1.5 dpa *lef1* was restricted to the most proximal BEL cells in control fins, while it was expressed as a domain lining the mesenchymal cells of each ray in denervated fins (Figure 5e’, f’). This was even more evident when assessing Lef1 protein accumulation at 2dpa (Additional file 3). Moreover, denervated fins that were regenerating at 1.5 dpa presented *lef1* mRNA expression spatially de-regulated, spread into the inter-ray region, suggesting that consecutive rays might have a continuous BEL (Figure 5g). Regarding the levels of expression, measured by qRT-PCR, *lef1* was decreased in denervated fins when compared with control fins (Figure 5y).

We have also investigated the expression of *wnt5b,* a non-canonical Wnt ligand that is usually expressed early in the WE and maintained in the distal BEL cells during fin outgrowth [6,11]. *Wnt5b* was expressed from 0.5 dpa to 4 dpa, both in denervated and control fins (Figure 5h-o), but qRT-PCR analysis showed an increment of 20% at 0.5 dpa in denervated fins, which was followed by a decrease from 1 to 2 dpa (Figure 5y). In cases where denervated fins were able to regenerate partially, *wnt5b* was expressed in a wider and irregular domain in the epidermis, suggesting a “merged blastema” with a common BEL in two consecutive rays (Figure 5m,m’,o).

Then, we analysed the expression of the Fgf target gene *pea3* and of *fgf24/wfgf*, to determine if the Fgf signalling in the regeneration epithelium was affected by denervation. The epidermal regulator *pea3,* which is expressed early in the WE and maintained in distal BEL cells during regenerative outgrowth [11], was expressed in a thin domain in the distal cells of the WE of denervated fins, at 0.5 and 1 dpa (Figure 5q’,s’), while it was expressed in the whole WE and inter-ray epidermis of control fins (Figure 5p’,r’). Additionally, analysis of qRT-PCR revealed a decrease in the levels of expression of *pea3* in denervated fins from 1 to 2 dpa when compared with control fins (Figure 5y). The ligand *fgf24,* which is usually expressed in the distal BEL cells during normal blastema formation and regenerative outgrowth [10,47], was also downregulated in denervated fins at 0.5 and 1 dpa (Figure 5y).

Similar to *pea3*, *krt8*, a cytokeratin expressed in the WE during the whole regenerative process [48], was strongly expressed in the whole WE of control fins (Figure 5t’,v’), while in denervated fins it was weakly expressed in some distal epidermal cells (Figure 5u’,x’). In addition, qRT-PCR revealed a decrease in *krt8* levels of expression in denervated fins (Figure 5y).

In summary, these results show that denervated fins were able to form a WE, thinner than the controls, but that expressed *fgf24, pea3* and *krt8,* as early as at 0.5 dpa and throughout later stages. Furthermore, the WE of denervated fins presented a BEL that expressed the usual markers *lef1* and *wnt5b,* but qRT-PCR analyses and mRNA or protein distribution showed that the patterns and relative levels of expression of these genes were remarkably altered in relation to controls. Overall, the above results suggest that denervation may cause functional and morphological changes in the AEC as well as interfere with the signalling pathways that coordinate its formation and maintenance.

### Blastema markers are altered on denervated fins

**Figure 6:**
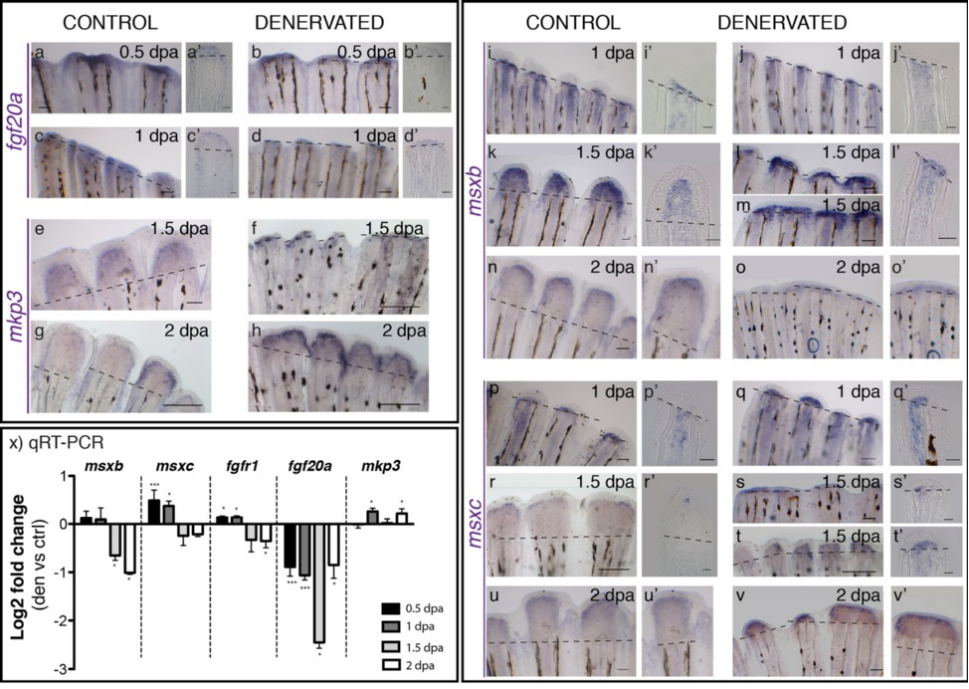
**Gene expression in the blastema of control and denervated fins. a-v)** mRNA ISH on whole mount amputated fins: *fgf20a, mkp3, msxb, msxc.* **a’-l’**, **p’-t’**) Longitudinal sections of the rays using whole mount ISH. **a-d)** *Fgf20a* expression is detected at 0.5 and 1 dpa in control fins **(a,c)** and only a residual expression is detected in denervated fins **(b,d)**. **e-k)** *Mkp3* is detected at 1.5 dpa in control **(e)** and denervated fins **(f)**. At 2 dpa the expression in denervated fins is reduced but stronger **(h)** than in controls **(g)**. **i-o)** *Msxb* expression starts at 1 dpa in both control and denervated fins. At 1.5 dpa denervated rays that have partially regenerated **(m)** present *msxb* expression spread in the inter-ray tissue (*). *msxb* expression is still detected at 2 dpa, however, in denervated fins is reduced to a thin domain **(o)**. **p-v)** *Msxc* expression is present in both fins at 1 dpa, but with a stronger expression in denervated fins (**q,q’)**. Expression is maintained after 1.5 dpa in non-regenerating **(s,s’)** and partially regenerating fins (**t,t’)**. At 2 dpa, *msxc* is expressed as a continuum in the tip of partially regenerating denervated fins, in what seems a “merged blastema” (Adult zebrafish pectoral fin denervation assay.,v’). **a,a’**-**v,v’)** Dashed lines mark amputation plane. Scale bar - 100 μm in whole mount fins; 25 μm in sections. **x)** qRT-PCR for genes expressed in the blastema. qRT-PCR analysis shows an increase in the expression of *msxc* and *fgfr1* on denervated fins at 0.5 and 1 dpa, as well as in *mkp3* at 1 and 2 dpa, when compared to controls. *Fgf20a* levels of expression are always decreased in relation to the controls, as it is *msxb* at 1.5 and 2 dpa. Note: ***p < 0.0001, *p < 0.05.

To investigate the impact of denervation in the signalling pathways that regulate blastema formation and outgrowth, we analysed the expression of several genes shown to play essential roles in those processes, namely, *fgf20a*, *msxb, msxc*, and *fgfr1.* The ligand *fgf20a,* a key regulator of fin regeneration, is expressed in the epithelial-mesenchymal boundary during wound healing, co-localizing later with *msxb* in the blastemal cells [49]. In denervated fins *fgf20a* presented a weak expression and qRT-PCR analysis showed a consistent decrease in its levels of expression from 0.5 to 2 dpa, when compared to controls (Figure 6a-d,x). By contrast, the expression of the Fgf inhibitor *mkp3* was increased in denervated fins at 1 and 2 dpa, in relation to controls (Figure 6e-h,x). The Fgf targets *msxb* and *msxc* are widely expressed in mesenchymal proliferating cells during caudal fin blastema formation and regenerative outgrowth [10,43,47,50]. Based on *in situ* hybridization analysis, our data showed that *msxb* mRNA started to be expressed at 1 dpa and it was maintained at 2 dpa, in both control and denervated fins (Figure 6i-o), however, a reduced and more proximal domain was observed in denervated fins (Figure 6j’,l’,o’). Analysis of qRT-PCR showed that the levels of expression of this gene were decreased at 1.5 and 2 dpa in comparison with control fins (Figure 6x). *Msxc* was also first detected by *in situ* hybridization at 1 dpa (Figure 6p,q). Although we could not detect *msxc* at 0.5 dpa by *in situ* hybridization, its levels of expression measured by qRT-PCR were higher in denervated than in control fins (Figure 6x). *Msxc* expression was still detected in control and denervated fins at 1.5 and 2dpa (Figure 6r-v) both in the non-regenerating (Figure 6s) and in the partially regenerating fins (Figure 6t,v). When denervated fins regenerated partially, *msxb* and *msxc* expression was spread in the inter-ray region, reinforcing that in some cases, consecutive rays formed “merged blastemas” with contiguous domains of gene expression (Figure 6v,v’). Similar to *msxc,* the expression of *fgfr1* was increased in denervated fins at 0.5 and 1 dpa, followed by a decrease at 1.5 and 2 dpa (Figure 6x). *Fgfr1* is usually expressed in blastema precursor cells underlying the WE during blastema formation, and it has been shown to control expression of *msx* genes in this process [10]. Overall, the above results showed that in denervated fins, the mesenchymal cells that give rise to the blastema exhibited significant changes in the expression of several markers. Denervation had a clear impact on Fgf signalling, and the most obvious effects were a reduction of the expression of the *fgf20a* ligand in all time points analysed, and an increased expression of *mxsc* and *fgfr1*, up to 1 dpa.

### Denervated fins exhibit scleroblast’s alignment defects

**Figure 7:**
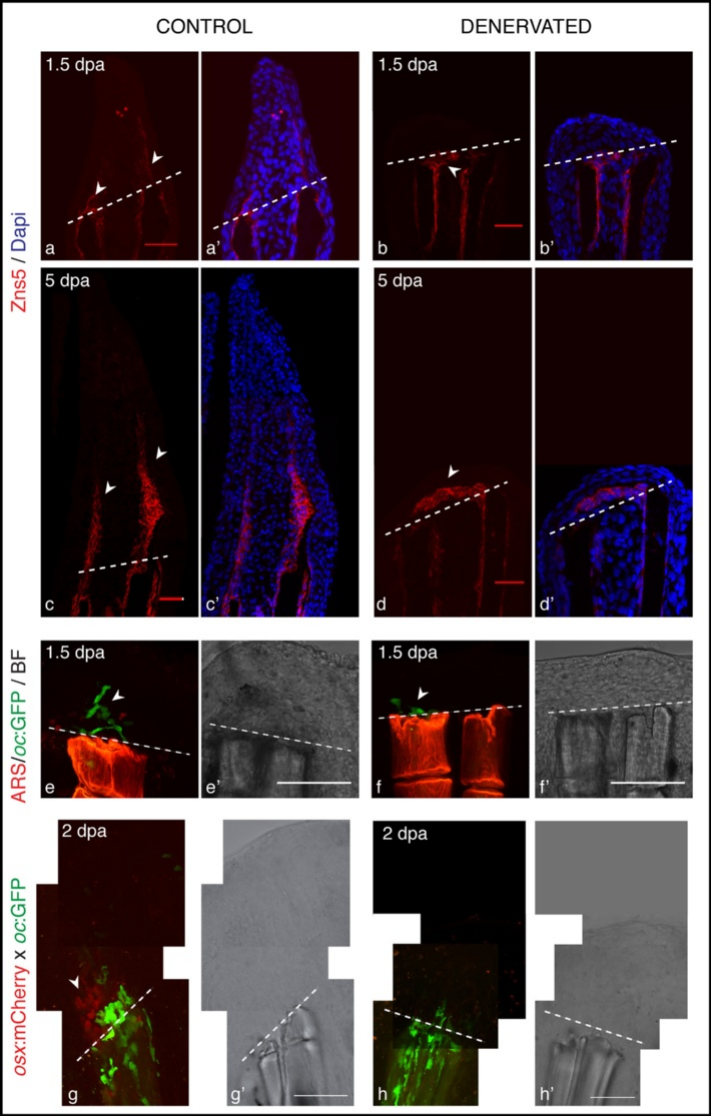
**Scleroblasts alignment in amputated fins, upon denervation. a-d)** Staining for Zns5 and DAPI in longitudinal sections shows that between 1 and 1.5 dpa, Zns5-positive cells start to accumulate just distal to the amputation plane in control fins (**a**-arrowhead). However, in denervated fins scleroblasts are not aligned with the stump rays, but instead are deposited between the 2 hemi-rays (**b**-arrowhead). At later time points the scleroblast deposition covers the tip of denervated rays **(d)**. **e,f)** Live imaging with the Tg (*oc*:GFP) co-stained with Alizarin red-S (ARS). *In vivo* imaging of Tg (*oc*:GFP) stained with ARS shows that mature bone cells (*oc-*positive) of control fins are localized at the amputation level and in the blastema (**e**-arrowhead). However, in denervated fins *oc-*positive cells do not migrate further than the amputation plane (**f**-arrowhead). **g,h)** Live imaging with the transgenics Tg (*oc*:GFP), Tg (*osx*:mCherry) and ARS. Zebrafish transgenic line resulting from an outcross between Tg(*oc*:GFP) and Tg(*osx*:mCherry) shows that at 2 dpa only control fins present *oc*-positive and *osx*-positive cells in the blastema (**g**-arrowhead). **a-h)** The images are a projection of confocal optical slices. Dashed lines mark amputation plane. **a-d)** Scale bar - 25 μm. **e-h)** Scale bar - 100 μm.

During normal fin regeneration, around 1 to 2 dpa, scleroblasts from the surrounding blastema start to align with the previously existing rays, along the BEL, to deposit bone matrix into the epithelial–mesenchymal interface, which will be progressively mineralized giving rise to the new bony structures [4,51,52]. The deposition of cells positive for Zns5, a marker for scleroblasts at various stages of differentiation [53], was observed in control fins just distal to the amputation plane aligned with the rays (Figure 7a,c). However, in denervated fins, Zns5-positive cells accumulating in the regenerating area did not align with the old bony rays, and instead started to deposit between the 2 hemi-rays at the site of amputation, below the BEL (Figure 7b). Later, in most cases, the accumulation of scleroblasts expanded and closed the ray, forming a “cap-like” structure in the tip (Figure 7d). In these fins, Lef1 was ectopically expressed in the most distal BEL cells (Additional file 4a’).

Whole mount and sectioned samples stained with Alizarin Red S dye, which labels calcium deposition in bone matrix [54], showed that the accumulation of scleroblasts was accompanied by deposition of new mineralized bone matrix (Additional file 4c), resulting in a thicker distal tip than the more proximal regions (Additional file 4e,e’). In addition, bright field images and morphological analysis of denervated fins at 4 dpa also confirmed that extracellular matrix was deposited between the 2 hemi-rays at the level of amputation (Additional file 4f-i).

The above observations raised several questions. Does the scleroblast accumulation in the stump of denervated fins originate from differentiated scleroblasts that have migrated from the stump to the amputation plane? Or does it originate from newly differentiated scleroblasts that arose from the mass of mesenchymal cells? To address these issues, we analysed the dynamics of differentiating scleroblasts, known to contribute to the blastema in control situations [4,55], using the zebrafish transgenic lines Tg(*oc*:GFP) [56] and Tg(*osx*:mCherry) [57], which express GFP and mCherry under the control of a *bglap*/*osteocalcin* (*oc*) and a *sp7/osterix* (*osx*) promoters, respectively.

Previous studies showed that differentiated scleroblasts from the stump, expressing the late bone differentiation marker *oc* [58], start to proliferate at 1 dpa, detach and migrate to the amputation plane, where they dedifferentiate to contribute to the blastema population [4,55]. Those scleroblasts, lose *oc* expression and re-express *osx,* a transcription factor present in early committed scleroblasts[4,55]. The present results show that *oc*-expressing cells, apparently migrating from the stump tissue, were localized at the amputation level of both control and denervated fins at 1.5 dpa (Figure 7e,f). However, in the controls, *oc*-expressing cells were spread throughout the blastema (Figure 7e), while in denervated fins, which have a residual or no blastema, remained at the amputation plane (Figure 7f), being possibly responsible for the thickening of ray tips (Additional file 4e). Furthermore, an outcross between the transgenics Tg(*Oc*:GFP) and Tg(*Osx*:mCherry) showed that at 2 dpa the early scleroblasts marker *osx* was only present in control fins (Figure 7g), indicating that in denervated fins scleroblasts did not dedifferentiate and re-express the early bone marker *osx*. This result suggests that the bone cells that have accumulated in the amputation plane (Figure 7F) arise from bone-differentiated cells (*oc*-positive) that have migrated to the site of injury remaining there, without dedifferentiating.

**Figure 8:**
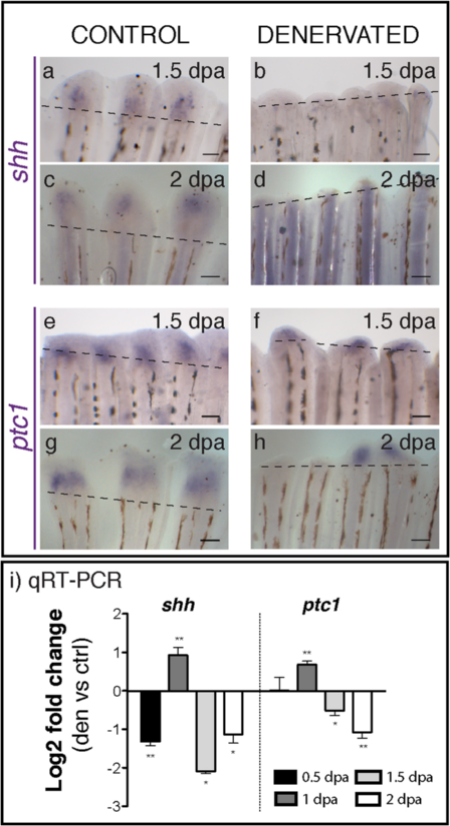
***Shh*** **and** ***ptc1*** **expression in control and denervated fins. a-h)** mRNA ISH on whole mount amputated fins. *Shh* expression is first detected at 1.5 dpa in the blastema of control fins **(a)**, but not in the denervated ones **(b)**. *Ptc1* mRNA starts to be expressed in the stump at 1 dpa both in control **(e)** and denervated fins **(f)**. While in control fins *ptc1* is expressed in every ray at 2 dpa, in denervated fins it is expressed only in the rays with a small blastema **(h)**. **a-h)** Dashed lines mark amputation plane. Scale bar - 100 μm. **i)** qRT-PCR for ***shh*** and *ptc1.* qRT-PCR reveals lower levels of *shh* expression on denervated fins in relation to controls, at 0.5 dpa. These levels abruptly increase at 1 dpa, decreasing again at 1.5 and 2 dpa. *Ptc1* expression is also higher at 1 dpa and lower at 1.5 and 2 dpa on denervated fins, in relation to controls (**p < 0.001, *p < 0.05).

In order to further characterise the effect of denervation in the spatial and temporal expression of genes involved in the deposition and alignment of new scleroblasts [59], we analysed the expression of s*hh* and its receptor *ptc1. Shh* mRNA started to be express in the blastema of control fins between 1.5 and 2 dpa (Figure 8a,c), but not in the denervated ones (Figure 8b,d). Although *shh* was never detected by *in situ* hybridization at 0.5 and 1 dpa in control or denervated fins, qRT-PCR showed that the levels of expression of *shh* were lower in denervated fins at 0.5 dpa, but in contrast abruptly increased at 1 dpa, decreasing again at 1.5 and 2 dpa (Figure 8i). *Ptc1* levels of expression were also higher at 1 dpa and lower at 1.5 and 2 dpa in denervated fins, in comparison with the controls (Figure 8i). *Ptc1* mRNA started to be expressed in the stump at 1.5 dpa and few differences were detected between control and denervated fins (Figure 8e,f), however, at 2 dpa denervated fins only expressed *ptc1* on the rays with a small blastema (Figure 8h). Together, these results indicate a de-regulation of Shh signalling during fin regeneration in consequence of nerve ablation, and subsequent de-regulation of genes involved in the differentiation of the newly secreting bone cells at 1.5 and 2 dpa.

### Anterior gradient proteins are expressed on regenerating fins

To further elucidate the molecular influence of nerves in zebrafish fin regeneration, we investigated the involvement of nAG, a secreted protein able to rescue a newt denervated blastema [34]. We started by addressing the expression of two nAG homologues: *ag1* [60] and *agr2* [60,61], during the regeneration of normal and denervated fins. *Agr2* expression has been previously reported during zebrafish development in several organs containing mucus-secreting cells, such as the epidermis, and in adult structures, such as the intestine [61]. However, the expression of *ag1* in zebrafish embryos and adults had not been described before.

Our results show that *agr2* mRNA was expressed in the mucus secreting cells of the epidermis of non-amputated fins (Additional file 5a), and maintained during all stages of regeneration in the entire epidermis of the fin, and also in the newly formed WE (Additional file 5c,e). *Ag1* exhibited a similar mRNA expression to *agr2,* during development [61], being present in the mucous secreting cells of the larval body at 48 hpf (Additional file 5 g). However, *ag1* was widely expressed in the epidermal cells in non-amputated and regenerating adult fins (Additional file 5 h,j), not specifically in the mucus secreting cells. Regarding nerve dependency, no differences were detected in the pattern of expression of both anterior gradient genes, between control and denervated fins (Additional file 5c-f,h-k). These results suggest that the Anterior Gradient protein family does not seem to play the same role during zebrafish fin regeneration as during amphibian limb regeneration.

## Discussion

**Figure 9:**
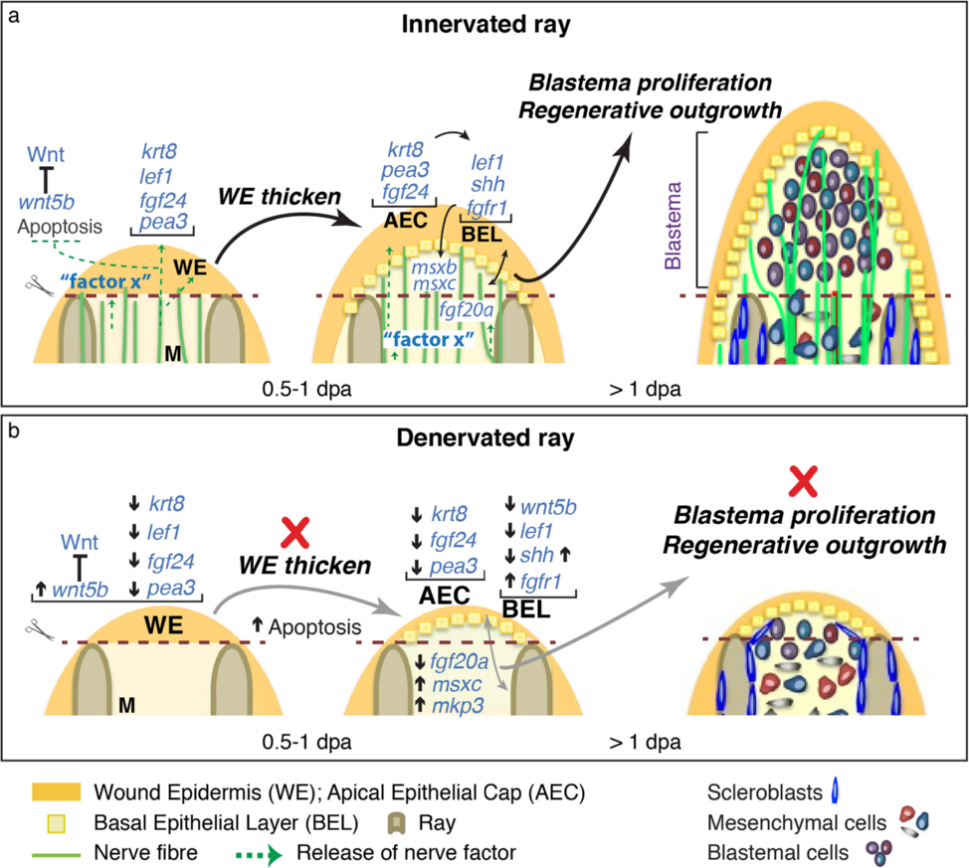
**Contribution of innervation to fin regeneration. a)** Illustration of the putative role of nerves in zebrafish fin regeneration. The WE is formed after fin amputation, in a process that is independent of nerves. Innervation may be important to the subsequent thickening of the WE and the establishment of the AEC, which contributes to the formation and outgrowth of the blastema and to the progression of the regenerative process. The role of nerves may be to release factor(s) (“factor x”) that regulate the expression of target genes in the WE, such as *pea3*, *fgf24* and *lef1*, which are important to the thickening and maintenance of the WE and to the communication established with the underlying cells. At the same time nerves may be involved in the inhibition of *wnt5b* in the WE, as well as in the pathways that lead to apoptosis. “Factor x” may also be directly released by nerves into the stump to induce cell proliferation and dedifferentiation. **b)** Illustration of the cellular and molecular changes occurred upon fin denervation. In the absence of innervation fins do not establish a functional AEC and several signalling pathways are affected. *Wnt5b*, an inhibitor of Wnt signalling and regeneration, is upregulated at 0.5 dpa, but is downregulated at 1 dpa. During this period krt8 and pea3, which are essential to WE maintenance, as well as fgf24 and lef1, important for the communication established with the underlying cells, are downregulated. At the same time, apoptosis activity is increased in the WE. The Fgf signalling molecules fgfr1, msxc, and mkp3 are upregulated, while fgf20a is downregulated. Shh is early downregulated at 0.5 dpa, but is then upregulated at 1 dpa. These signalling defects result in a breakdown of communication with mesenchymal cells, and impairment of the formation of the blastema and fin regeneration.

In this report we describe an efficient protocol for zebrafish pectoral fin denervation, which allowed us to show that zebrafish fin regeneration is dependent on nerve supply and that the activity of several factors, known to be required for fin regeneration, is affected by fin denervation (Figure 9). Following amputation, denervated fins were able to close the wound and establish a WE within the first hours. However, the thickening of the WE and consequent formation of the AEC were severally affected by denervation, suggesting that the presence of nerve fibres in the site of injury is required immediately after wound closure. In the complete absence of nerve fibres in the stump, denervated rays were not able to establish a blastema and regeneration was impaired. However, in the presence of a reduced amount of nerves in the stump, a residual blastema was still formed and fins exhibited defective regeneration, giving rise to smaller and abnormal fins. The observation that rays with less innervation also showed less regeneration, suggests that there is a dependency on the quantity of innervation to fin regeneration efficiency. This quantitative requirement has been previously described in amphibians and in other Teleosts, where a certain amount of innervation must be met for regeneration to be successful [62,63].

Another consequence of nerve ablation was the narrowing of denervated fins, which has been previously observed in the *Fundulus heteroclitus* experiments [18]. We were not able to conclude that apoptosis was responsible for this effect, since we did not observe caspase-3 activity in the entire inter-ray tissue, but only in its distal tip. Therefore, we can only speculate that narrower denervated fins were a consequence of changes in tissue morphology that results from the absence of nerve fibres, or alternatively that the absence of fin movement observed after nerve ablation leads to subtle changes in the fin structure at the level of the inter-rays.

We have also observed a reduction in the size of the regenerates when denervation was performed at 1 dpa, during the formation of the blastema. However, when denervation was performed after the formation of the blastema we could not detect a significant difference in fin outgrowth between denervated and control fins. Nevertheless, denervated fins were narrower than the controls and presented crooked rays, suggesting a less essential role in tissue morphogenesis during outgrowth. In amphibians, similar observations were obtained and the extent of regeneration impairment was shown to be dependent on the time point of limb denervation [17,21,64]. These results indicate that nerves are fundamental in the initial stages of the regeneration course, controlling the first steps of wound healing and formation of the blastema, the driving force of the all process, but that they also exert some effect in tissue morphogenesis upon regenerative outgrowth.

In order to understand the specific effect of denervation in each step of the regeneration process, we have analysed key molecular pathways associated with the establishment and maintenance of the WE and blastema. The WE of denervated fins were consistently thinner than the controls and did not develop into a fully functional AEC. Our observations of caspase-3 activity suggest that this was associated with the increased apoptosis observed in the epidermis of denervated fins. In addition, although the epidermal markers *krt8*, *lef1* and *wnt5b* could still be detected in the WE of denervated fins, the levels and domains of expression were abnormal, which is a possible explanation for the observed defects. We can hypothesize that low levels of *krt8* may contribute to the failure in the establishment of a functional WE which consequently may lead to changes in gene expression, since Keratins are required for the tensile strength of keratinocytes [65], and have been proposed to play roles in stress response, cell signalling and apoptosis [66]. In the case of *wnt5b*, the upregulation in amputated denervated fins may be due to the suppression of an inhibitory mechanism that is normally activated by nerves. In turn, this *wnt5b* increased expression in denervated fins, contributes to explain regeneration impairment and *lef1* downregulation. This hypothesis is based on previous work showing that high levels of *wnt5b* inhibit blastema proliferation and regenerative growth, by reducing the expression of Wnt/β-catenin target genes, such as *lef1*, during fin regeneration [6]. On the other hand, the ectopic expression of Lef1 in distal BEL cells in denervated fins might be related with the later downregulation of *wnt5b* observed after 1dpa, as during normal fin regeneration *wnt5b* expression in the distal BEL cells restricts *lef1* expression domains [11]. It is worth to note that the correct levels of Wnt signalling need to be precisely controlled during regeneration. Wnt signalling has been proposed to orchestrate fin regeneration [13] and overexpression of a Wnt/ßcatenin signalling inhibitor (Dkk), resulted in improper specification of the WE, with lower levels of *lef1*, and also in downregulation of the blastema markers *msxb* and *fgf20a* [6,67]. Such reduction in gene expression caused by Wnt signalling inhibition is similar to the reduction in expression of Wnt targets in the blastema of denervated fins.

Our results also showed that a number of other genes that are essential players during blastema formation, namely *msxb*, *msxc*, *fgfr1* and *fgf20a* were significantly altered in denervated fins. The consistent low levels of expression of the ligand *fgf20a*, which is required for fin regeneration initiation and blastema formation/proliferation [49], might be responsible for the failure of correct blastema formation and outgrowth, while low levels of *msxb* at 1.5 and 2 dpa may be an indirect consequence of the lack of blastema. We can speculate that *fgfr1* upregulation might be part of a compensatory mechanism induced by the downregulation of Fgf signalling in the absence of nerves. The increased expression of this receptor at early time points might then induce the early upregulation of *msxc*, which has been shown to act downstream of *fgfr1* during fin regeneration [10,68,69]. Also, the upregulation of the Mapk phosphatase, *mkp3,* in denervated fins might reflect a compensatory mechanism induced by the downregulation of Fgf signalling. Mapk is a highly conserved pathway involved in cell proliferation, differentiation and migration [70,71] that is both a downstream target and an inhibitor of the Fgf pathway [72]. In experiments where this pathway was inhibited after fin amputation, a WE was formed and the initial disorganization and migration of mesenchymal cells proximal to the level of amputation still occurred [10,68,69], but regeneration was blocked, similarly to what we show in the absence of nerves. In summary, our data strongly suggest that Fgf signalling is involved in the contribution of nerves to fin regeneration.

The present results indicate that denervation caused molecular, functional, and morphological changes in the blastema and regenerating epithelium. The lack of innervation resulted in a failure to accumulate additional epithelial layers and to coordinate the correct BEL signalling events, compromising the communication with the underlying mesenchyme. It is important to stress that the secretory activity of the BEL is crucial to the epithelial-mesenchymal communication that assures the success of blastema formation and outgrowth [73]. If nerves exert their function through targeting the AEC, which then signals to the underlying mesenchymal cells inducing cell dedifferentiation and proliferation, the formation of a defective AEC certainly affects this process. Indeed, our results showed that although mesenchymal cells underlying the WE of denervated fins became disorganized, they failed to form a proliferative blastema and regenerate. The alterations on the expression of cell cycle progression markers, together with the initial increase of *mps1* on denervated fins, suggests that these cells were able to enter the cell cycle, but were then arrested in the G/M transition, blocking blastema growth. These results are in accordance with previous studies in denervated amphibians limbs showing that tissue dedifferentiation could still occur upon amputation of denervated limbs and a normal percentage of cells re-entered the cell cycle. However, most of those cells failed to progress through the S phase and the blastema did not form [19,21,74,75].

Besides the clear role of nerves in the WE and blastema formation we were surprised to observe that scleroblast activity in the regenerating area was not completely impaired, but instead led to ectopic deposition of bone matrix in the ray stump forming a “cap-like” mineralized structure. These observations led to the analysis of Shh pathway, known to control scleroblasts differentiation, alignment, and proliferation during fin regeneration [10,12]. We were able to see that *shh* expression was altered in denervated fins, being decreased at 0.5 dpa and then increased at 1 dpa together with its target gene *ptc1*. Previous work has shown that upon *shh* inhibition during fin regeneration, blastema proliferation is arrested [11], and *shh* overexpression results in ectopic bone deposition and abnormal patterning [59]. Additionally, in the absence of a properly formed blastema, the BEL cells may direct this factor into areas where it is normally not secreted, leading to incorrect scleroblast’s alignment. We can also speculate that the increment in *shh* expression observed in denervated fins at 1 dpa was due to the increase in *fgfr1*, as Fgf signalling has been proposed to be required for normal expression of *shh* in the fin regenerate [10,11].

Previous work in amphibians has suggested that nerves exert their function by releasing specific factors crucial for the activation and maintenance of the regeneration process [24,25,28,76]. In zebrafish, the requirement of innervation during fin regeneration also seems to depend on the release of factors (“factor x”) that target the WE and/or are directly secreted into the stump (Figure 9). In amphibians, Fgfs [24-27], neuregulin [29,30], GDF5 [26], transferrin [31,32] and substance P [33] have been suggested to play the role of such factors. In newts, nAg has been proposed to be the nerve-secreted factor that underlies regeneration dependency on nerves [34]. We have explored a parallel function in zebrafish, but our results suggest that the Anterior Gradient (Ag) protein family do not play the same role as in newts. This is not completely unexpected, as newts seem to have evolved species-specific interactions that are not present in Teleosts. In newts, nAG interacts with the cell surface determinant of positional identity, Prod1, a salamander-specific member of the three-finger protein (TFP) family, with no obvious homologues in other vertebrates [34,77]. nAG activity in other vertebrates would require another receptor, possibly a taxon-specific TFP, in place of Prod1 [77]. This interpretation is in line with the hypothesis that regeneration results from multiple, independent evolutionary origins. In this scenario, the regeneration of salamander’s limbs has evolved locally and it is regulated at least in part by taxon-specific components, which in salamander involved the expansion of the TFP family [77]. Moreover, recent phylogenetic analysis of Ag proteins has shown that fish *agr2* is more similar to Agr2 of higher vertebrates than amphibians [60]. Thus, it is possible that trophic factors released by nerves have followed alternative evolutionary routes in different species. It is also conceivable that nerves secrete more than one trophic factor and that there are both different and common factors across species.

In zebrafish, it is conceivable that Ffgs, Wnts, or Shh are secreted by fin nerve fibres, as these factors have been shown to be produced by nerves in other systems [78-80], and the effects of manipulating their levels during fin regeneration have some similarities with the effects of fin denervation. In the case of Fgfs, there are reports in amphibian limb regeneration proposing such role [24-28], but in zebrafish fins it is unlikely that Fgf is the factor released by nerves since its downstream targets, *msxc* and *mkp3* were increased in denervated tissue. Also, our results do not support the hypothesis of *shh* playing the role of ”factor x”, because in the absence of innervation, its target, *ptc1,* is only affected significantly after 1 dpa. Regarding Wnts, there are no previous indications that any of the members of this family can be released by nerves in the context of regeneration, but our data does not rule it out. Both in denervated fins and in Wnt loss of function experiments [7], the target *lef1* is significantly reduced and the establishment of the WE and its subsequent thickening is severely compromised. Taken together the above results indicate that contribution of nerves to regeneration involves the regulation of Ffg, Wnt and Shh signalling pathways. However, the effects of nerve absence seem to be more complex than the disruption of any of the pathways alone and may reflect combined effects of multiple signalling cascades.

## Conclusions

This study contributes to establishing the zebrafish pectoral fin as an *in vivo* valuable model to understand the molecular and cellular mechanisms of nerve dependence during vertebrate regeneration. We have shown, for the first time, that zebrafish fin regeneration is dependent on proper nerve supply. Our results indicate that denervation produces a dysfunctional attempt at regeneration: in the absence of innervation fins are able to form a thin WE, but do not establish a functional AEC and proliferation of blastemal cells is not sustained (Figure 9). Defects in the AEC are detrimental to regeneration since this structure establishes a crucial communication with the underlying mesenchyme to promote the proliferation of blastemal cells. We show that factors known to play a role in regeneration, including members of the Fgf, Wnt and Shh signalling cascades, are affected by denervation suggesting that these signalling factors mediate the action of nerves. The role of nerves may be to release a “factor x”, which interferes with the expression of those signalling pathways, directly or indirectly, to produce the appropriate expression levels of the factors that are required for a productive regenerative process.

## Methods

### Fin denervation and amputation assay

Wild-type AB strain zebrafish (*Danio rerio*) of 3–12 month old were used in all experiments. Animals were maintained in 28°C water tanks under standard conditions [81]*.* Only female individuals were used, since AB males have defective pectoral fin regeneration and present characteristic epidermal ornamentations in the ventral surface of the fin that hinder satisfactory microscopic observations (personal observation and [82,83]). For all procedures, fish were anesthetized in water containing 4 mg/ml Tricaine (MS-222, Sigma). The right pectoral fin was denervated by surgically resecting the nerves at the level of the brachial plexus (Figure 1a,b), assuring that the blood vessels that run along with nerve fibres were not resected as well and there was no blood leakage. The left fin was used as the innervated control. In the next day, the right fin was re-denervated to assure efficient nerve degeneration. After 6–8 hours post re-denervation (sufficient time for nerve retraction and degeneration - data not shown) both fins were amputated below the level of the first ray bifurcation, using surgical scissors. At this point, the discarded tissue was collected for immunofluorescence using the axonal marker ac. α-tub to check nerve presence/absence (Figure 1c,d). Fish were then placed in 33°C tanks, temperature that accelerates regeneration [53], and regeneration was allowed to proceed. Re-denervation took place every day after amputation, to avoid nerve recovery, which was observed to occur approximately 2 days post denervation. After several time points post amputation regenerated fins were collected for further analysis. In order to discard the possibility of a heat-shock effect in the consequences of nerve ablation, we have also performed a set of denervation experiments at 28°C. As the results were similar at both temperatures we have chosen to conduct all experiments at 33°C. We have also performed an experiment where fins were denervated after amputation (at 1 or 2 dpa). In this case fins were collected and measured after 3 or 4 days post amputation. Experimental procedures with zebrafish were performed according to European Union directives and Portuguese law (Directive 2010/63/EU, Decreto-Lei 113/2013) and approved by the Animal User and Ethical Committees at Instituto Medicina Molecular and Instituto Gulbenkian Ciência. Reporting conforms to the Animal Research: Reporting In Vivo Experiments (ARRIVE) guidelines.

### Immunofluorescence on whole mount tissues

Pectoral fins were fixed overnight (o/n) at 4°C in a solution of 20% DMSO (Sigma, #D8418) in Methanol (MetOH). The following day, fins were rehydrated in MetOH/PBST0.3 (0.3% Triton X-100 in PBS-phosphate buffered saline) series, and then permeabilized with acetone for 20 minutes (min) at −20°C. After several washes with PBST0.3, fins were included for at least 1 hour in blocking solution (1% BSA, 1% goat serum, 1% DMSO in PBST0.3) at room temperature (R.T.). Incubation with primary antibody (ab.), diluted in blocking solution, took place o/n at 4°C. Non-conjugated antibody was removed by several washes with PBST0.3 and appropriate secondary alexa fluor ab. (Molecular Probes) was diluted in blocking solution, and left o/n at 4°C in the dark. Several washes with PBST0.3 were performed to remove the excess ab., and samples were counterstained with 0.15% (w/v) DAPI (4',6-diamidino-2-phenylindole, Sigma #D9564) in PBS, for 30 min. At the end, fins were washed in PBS and stored in mounting medium (2% DABCO, Sigma #D2522 and 80% Glycerol in PBS) for microscope analysis.

### Immunofluorescence on cryosections

Pectoral fins were fixed o/n at 4°C in a solution of 20% DMSO in MetOH and processed for 8 μm cryosections. After defrosting, slices were washed twice with PBS at 37°C for 10 min, followed by 20 min permeabilization with PBST0.3 at R.T. After several washes in PBS, sections were blocked in 10% Fetal Bovine Serum (FBS) in PBS for at least 1 hour, and incubated with primary ab., o/n at 4°C. Several washes with PBST0.3 were performed to remove non-conjugated ab. and appropriate secondary alexa fluor ab. was diluted in blocking solution and incubated o/n at 4°C, in the dark. All sections were counterstained with 0.15% (w/v) DAPI in PBS for 15 min, washed in PBS and mounted in Dako fluorescent mounting media (Dako, #S3023), for microscope analysis.

The primary antibodies used were the following: Mouse monoclonal anti-Acetylated α-Tubulin antibody (Sigma, #T7451); Mouse monoclonal anti-P63 antibody (Santa Cruz, #sc-8431); Rabbit polyclonal anti-active Caspase3 antibody (Abcam, #Ab13847); Rabbit polyclonal anti-Tenascin C (US Biological, #137.T2550-23); Rabbit polyclonal anti-PCNA antibody (Santa Cruz Biotech, #SC-7907); Rabbit polyclonal anti-Phosphohistone H3 (H3P) Milipore, #06-570; Rabbit polyclonal anti-Lef1 antibody (Abmart, #P30013); Mouse monoclonal Zns5 (ZIRC, #011604).

### Measurements and quantifications

Regenerate area, length and width were measured using *ImageJ* software. For each fin, all ray lengths and areas were measured from the amputation plane until the most distal tip. For the width, the length between 2 consecutive rays was measured. For Caspase-3 and 3HP we quantified the number of positive cells normalized against the tissue area. Paired *t*-test was used to compare all sample means, except for those with non Gaussian population, where a Wilcoxon matched-pairs signed rank test was performed. p values <0.05 were considered to indicate statistical significance. Graphics and statistical analysis were performed using *GraphPad Prism* version 5.00 for Mac, GraphPad Software, San Diego California USA.

### Histology

The fins were fixed in 2.5% glutaraldehyde solution in PBS for 3 hours, washed extensively in PBS and dehydrated in ascending series of EtOH. The tissues were embedded in Historesin (Leica microsystems) plastic embedding medium following manufacturer’s protocol. The sections were cut at 2 μm on an automatic microtome (*Leica RM 2155*) using a tungsten carbide knife. The sections were stained with Toluidine blue [84], air dried and cover slipped using DPX mountant.

### Whole-mount *in situ* hybridization (ISH) on zebrafish adult fins

Fins were fixed o/n in ice cold 4% formaldehyde in PBS, dehydrated in a gradient of MetOH/PBS at R.T., and stored in 100% MetOH at −20°C until processed. Fins were rehydrated in MetOH/PBT (0.1% Tween20 in PBS) series. Inactivation of endogenous peroxidases was performed for 10 min by using a solution of 6% H_2_O_2_ in PBT, which was then removed by washing twice in PBT for 5 min. Fins were digested in 10 mg/ml Proteinase K (Sigma, #P6556) in PBT for 25 min and then re-fixed in 3.7% formaldehyde plus 0.2% glutaraldehyde in PBT, for 20 min. After washing in PBT, pre-hybridization took place for at least 1 hour at 70°C, in hybridization solution (60% formamide, 5× SSC (pH 6.0), 500 μg/ml tRNA, 0,1% Tween20, 50 μg/ml heparin, in Rnase free MQH_2_O). Hybridization (hyb) took place o/n at 70°C in hyb solution, containing 5 μl/ml digoxigenin-labeled RNA probe*.* In the next day, several washes at 70°C with hyb solution and SSC, removed the un-hybridized probe*.* Fins were then washed in a gradient of SSC0.2x/PBT and then in TBST (Tris-Buffered Saline, 0.1% Tween 20). Finally, samples were pre-incubated in blocking solution (10% goat serum in TBST) at R.T. for at least 1 hour. Incubation with 1:2000 anti-digoxigenin antibody coupled to alkaline phosphatase (Roche, #11093274910) in blocking solution took place o/n at 4°C in the dark*.* In the next day several washes with tetramisole hydrochloride (Sigma, #L9756) in TBST, removed the uncoupled antibody. The alkaline phosphatase reaction was performed by 3 changes on reaction buffer NTMT (5 M NaCl, 1 M Tris HCl pH 9.5, 1 M MgCl_2,_ 10% Tween20, in H_2_O MQ). Reaction development was done with 1 μl/ml NBT (4-nitro blue tetrazolium chloride, Roche #11383213001) and 3.5 μl/ml BCIP (5-bromo-4chloro-3-indolyl-phosphatase, Roche #1383221) in NTMT, and stopped with several washes of PBT. At the end fins were photographed and processed for cryosectioning (12 μm). ISH on zebrafish embryos was performed as previously described [85]. DIG-labelled antisense RNA probes for all studied genes were synthesized as described by Henrique *et al*. [86].

Gene products were cloned either by PCR amplification of zebrafish cDNA, or by EST clones. The following were used: *ag1* (Fw: TGATCATTCATCATTTGGAGGA; Rev:TTACAGATCATCATGTTCCTCGTG); *krt8* (Fw:ATGTCCACCTACAGCAAGAAAAC; Rev:TCAATCTTGGACTACTTCAGAGGAC); *agr2* (full length cDNA clone [IRBOp991A0567D]); *lef1* (EST clone [ImaGenes998C 1015213Q1]); *pea3, msxb,* and *msxc* were kindly provided by Henry Roehl’s Laboratory; f*gf20a* was kindly provided by Kenneth Poss’s Laboratory; *wnt5b* and *mkp3* were kindly provided by Leonor Saúde’s Laboratory.

### Alizarin staining

Fish were immersed in a 0.01% ARS solution for 15 minutes and washed in water prior to live imaging.

### Imaging

Histology and *in situ* hybridization pictures were captured with a *Leica Z6APO* stereoscope equipped with a *Leica DFC490* digital camera, and a *Leica DM2500* microscope equipped with a *Leica DFC420*. Fluorescent images were acquired with a *Zeiss LSM 710* confocal microscope.

### RNA extraction

Total RNA was extracted from control and denervated pectoral fins from 4 different time points (0.5 dpa, 1 dpa, 1.5 dpa, and 2 dpa) using TrizolTM (Invitrogen), and were treated with *DNase set* from the *Qiagen RNeasy Micro kit* (Qiagen, Hilden, Germany) according to the manufacturer’s protocol. RNA quantity was determined by spectrophotometry using *Nanodrop 2000c* equipment (Thermo Scientific). Replicates of 10 fins were used for each sample for all time points analysed.

### qRT-PCR

For qRT-PCR analysis, first-strand cDNA was synthesized from 0.5 μg of total RNA using *Superscript II Reverse Transcriptase* (Invitrogen) with random hexamers. After synthesis, each cDNA sample was diluted 5-fold and used in PCR reaction with gene-specific primers (Table 1). The absence of contaminating genomic DNA was confirmed for each RNA extraction by PCR amplification of β*-actin* specific product from RT negative samples. Quantitative PCR reactions were carried out in an *Applied Biosystems 7500 Fast Real-Time PCR* System using a 20 μl volume containing 10 μl *Itaq Fast SYBR green* (Bio-Rad 172–5101); 4 μl of diluted cDNA and 0.2 mM of each primer. Reaction performed: 2 min at 50°C and 5 min at 95°C, 40× (10 seconds at 95°C; 30 seconds at primer specific temperature - Table 1). The specificity of the reactions was confirmed by using melting curve and gel electrophoresis analysis. Control assays containing no cDNA were also performed. 2-DDCt method was used to calculate the expression levels of each gene in relation to control. Results were subsequently analysed for statistical significance using a *t-*test or a Wilcoxon matched-pairs signed rank. p values <0.05 were considered to indicate statistical significance. Graphics and statistical analysis were performed using *GraphPad Prism version 5.00* for Mac, Graph Pad Software, San Diego California USA. The relative amount of each transcript was normalized to the level of the housekeeping gene β*-actin*. The primers were designed using the software *Custom Primers - OligoPerfect™* Designer (Invitrogen), and are indicated in Table 1.

**Table 1:** **Gene specific primers used in qRT-PCR experiments**

**Gene**	**Primers**	**Temperature**
β*-actin*	Fw: GCTTCACCACCACAGCCGAAAGA;	60°C/63°C
	Rev: GATACCGCAAGATTCCATACCCAGG	
*lef1*	Fw: AAGGCCACCCGTACCCGAGT;	60°C
	Rev: GGGTGAACGGCATGGGACGG	
*pea3*	Fw: GCCTGGCTGCCCATCCATGT;	63°C
	Rev: AATTCCATGCCACGGCCCGT	
*wnt5b*	Fw: TTGACGGACAAGCTGTTCAACCAA;	63°C
	Rev: ACCACCACGAGTTGGCGACC	
*krt8*	Fw: CGGTCTTGGCATGGGCATGGG;	63°C
	Rev: TGGAGCGTGTGGCTGTCTGGT	
*fgf24*	Fw: CGCCACTTACTGGAGCGGCAA;	63°C
	Rev: GGCTCACGTCGTCTCGAGTG	
*msxb*	Fw: CCAGCAGGTCGCGTGTTCTCC;	60°C
	Rev: GCTTGCGTAAGGTGCACGGC	
*msxc*	Fw: AGGGACAGTCCGGCTGGTTTCA;	60°C
	Rev: ACTGCGAGGTGGTAAACGGGG	
*mkp3*	Fw: GGTTCGCGCGGAGATGCAAGA;	63°C
	Rev: CCCTCCGAGACCCAGGACCTG	
*fgf20a*	Fw: GGTTCGGTCCAAGGCACGAGG;	63°C
	Rev: CGCTCGCCATGCCGATACAGG	
*fgfr1*	Fw: ACACGCCTGCGCAACGATCA;	63°C
	Rev: GTTGAGCCCAGACGGGTGCC	
*mps1*	Fw: ACCAGTAGGGAGCACGCGCT;	63°C
	Rev: GGCAGGTGTCCGGGGAGTTTG	
*shh*	Fw: GGCCAGGGGTTAAGCTGCGT;	63°C
	Rev: CGGCCTTCTGTCCTCCGTCC	
*ptc1*	Fw: TCTGCAAGCCACTTTTGATG	63°C
	Rev: AGGATGGGGGTAAAAGTTGG	

## Additional files

Additional file 1:
**Analysis of the regenerative process of denervated pectoral fins.** a-d) Staining for ac. α-tub and DAPI in whole mount fins. Staining with DAPI confirms that fins without innervation (b,d) have a WE with less epidermal cell layers than controls (a,c). e-h) Staining for ac. α-tub and DAPI in sections. Staining with DAPI shows that fins without innervation (e) have a WE with less epidermal cell layers than controls (f). After 1.5 dpa denervated fins (h) are not able to regenerate as the controls (g). a-h) Dashed lines mark amputation plane. i,j) Quantification of the length of regenerated tissue in fins denervated after amputation. Measurements of the length of regenerated tissue, taken from the amputation site to the most distal tip. i) There is a consistent significant reduction (***p < 0.0001) in the length of fins denervated at 1 dpa in relation to controls, both at 2 and 3 dpa. j) There is no significant reduction in the length of regenerates in fins denervated at 2dpa in relation to controls (fix at 3 dpa p = 0.33; fix at 4 dpa p = 0.07). Additional file 2:
**Mesenchymal tissue disorganization after amputation of denervated fins.** a,b) Staining for Tenascin C and ac. α-tub in whole mount fins at 0.5 dpa. Tenascin C is present in the mesenchymal tissue under the amputation plane, both in control and denervated fins. c,d) Toluidine Blue histology in longitudinal sections. Longitudinal sections stained with toluidine blue show that at 1 dpa mesenchymal tissue becomes more disorganised below the amputation plane, with cells presenting a more elongated shape that suggests cell migration, in both control (c*) and denervated fins (d*), while more proximal mesenchymal tissue presents a more organized structure (c**, d**). a-d) The images are a projection of confocal optical slices. Dashed lines mark amputation plane. a,b) Scale bar - 100 μm. c,d) Scale bar - 50 μm. Additional file 3:
**Staining for Lef1 in longitudinal sections after amputation of denervated fins.** Lef1 protein is detected in the BEL of both control and denervated fins from 0.5 to 2 dpa. At 0.5 dpa Lef1 expression is similar in control (a) and denervated (b) fins. At 2 dpa Lef1 is restricted to the most proximal BEL cells in control fins (c-arrowhead) and it is expressed all over the BEL lining mesenchymal cells of denervated fins (d-arrowhead). Scale bar - 25 μm. a-d) The images are a projection of confocal optical slices. Dashed lines mark amputation plane. Scale bar - 25 μm. Additional file 4:
**Bone deposition in the amputation plane of denervated fins.** a) Staining in longitudinal sections for Zns5, Lef1 and DAPI. Zns5-positive cells accumulate below the BEL of denervated fins, forming an arch that delimits mesenchymal cells. Lef1 is ectopically expressed in the whole BEL (a’). b,c) Staining with ARS in longitudinal sections. Alizarin Red staining at 6 dpa shows mineralization of the newly formed ray along the old bony ray in control fins (b-arrowhead), while in denervated fins it shows bone deposition in at the amputation level (c -arrowhead). d,e) Staining with ARS in whole mount fins. The deposition of bone cells in the amputation site causes a thickening in the distal stump of deneravated fins. A 3D projection of denervated rays shows that the distal region (e-arrowhead) is comparatively thicker in relation to the proximal region (e’-arrowhead). f-h) Toluidine Blue histology in longitudinal sections. At 4 dpa cell deposition between the 2 hemi-rays is observed in denervated fins (g,h). The new structure resembling bone matrix seems to be derived from the old bone (g - arrowhead) and a group of cells, resembling bone cells, are deposited at the level of amputation almost closing the ray (h - arrowhead). i) DAPI staining in longitudinal sections. At 6 dpa a bright field image shows matrix deposition at the level of amputation between the two hemi-rays (arrowhead). a-e, i) The images are a projection of confocal optical slices. Dashed lines mark amputation plane. a,e) Scale bar - 25 μm. b-d) Scale bar - 50 μm. Additional file 5:
**Expression of nAg homologues in zebrafish.** a-f) Whole mount mRNA ISH of *agr2*. c’-f’) Longitudinal sections of the rays using whole mount ISH. *Agr2* mRNA is expressed in the mucous secreting cells of the entire epidermis in both non-amputated (a,b) and amputated fins (c-f). During regeneration, *agr2* is also expressed in the mucous secreting cells present in the newly formed WE. There are no obvious differences between control (a,c,e) and denervated fins (b,d,f) in the amount of *agr2-* positive cells, in both non-amputated fins and during regeneration. g-k) Whole mount mRNA ISH of *ag1*. g) *Ag1* mRNA is expressed in the mucous secreting cells of the larvae body at 48 hours post fertilization (hpf). h-k) Longitudinal sections of the rays using whole mount ISH show wide expression of *ag1* in the epidermis and WE of adult fins, as early as 0.5 dpa, and throughout regeneration. There are no differences in *ag1* expression between control and denervated fins, in both non-amputated and amputated fins.

## Abbreviations

BEL: Basal epithelial layer
WE: Wound epidermis
ISH: *in situ* hybridization
BF: Brightfield
dpa: Days post amputation
