# Supplementary figures and images for: Denervation impairs regeneration of amputated zebrafish fins
Source: BMC Dev Biol. 2014 Dec 31;14:49. doi: 10.1186/s12861-014-0049-2 (PMC4333893; doi:10.1186/s12861-014-0049-2)

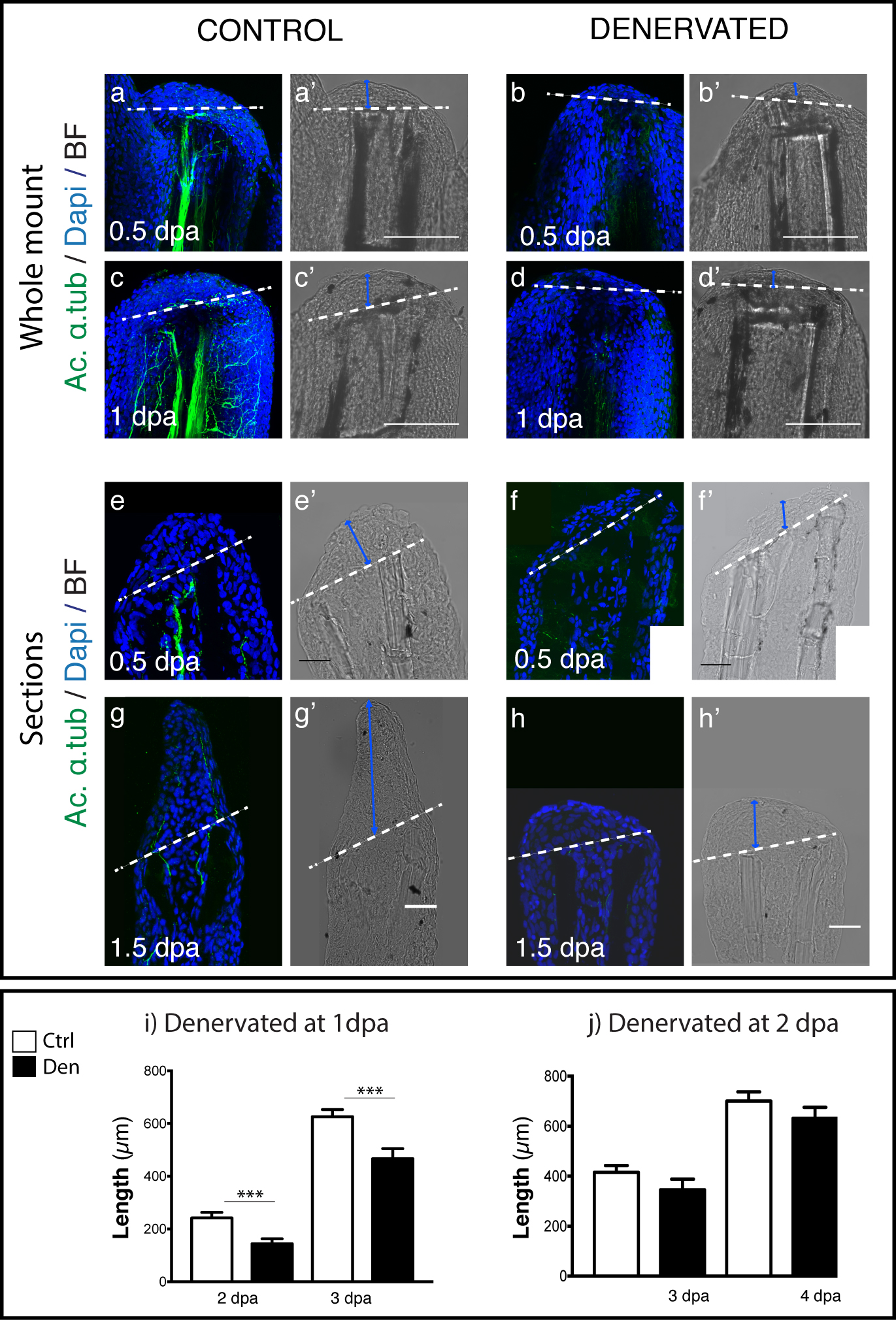

Supplement: Additional file 1: — Analysis of the regenerative process of denervated pectoral fins. a-d) Staining for ac. α-tub and DAPI in whole mount fins. Staining with DAPI confirms that fins without innervation (b,d) have a WE with less epidermal cell layers than controls (a,c). e-h) Staining for ac. α-tub and DAPI in sections. Staining with DAPI shows that fins without innervation (e) have a WE with less epidermal cell layers than controls (f). After 1.5 dpa denervated fins (h) are not able to regenerate as the controls (g). a-h) Dashed lines mark amputation plane. i,j) Quantification of the length of regenerated tissue in fins denervated after amputation. Measurements of the length of regenerated tissue, taken from the amputation site to the most distal tip. i) There is a consistent significant reduction (***p < 0.0001) in the length of fins denervated at 1 dpa in relation to controls, both at 2 and 3 dpa. j) There is no significant reduction in the length of regenerates in fins denervated at 2dpa in relation to controls (fix at 3 dpa p = 0.33; fix at 4 dpa p = 0.07). [file 12861_2014_49_MOESM1_ESM.jpeg]

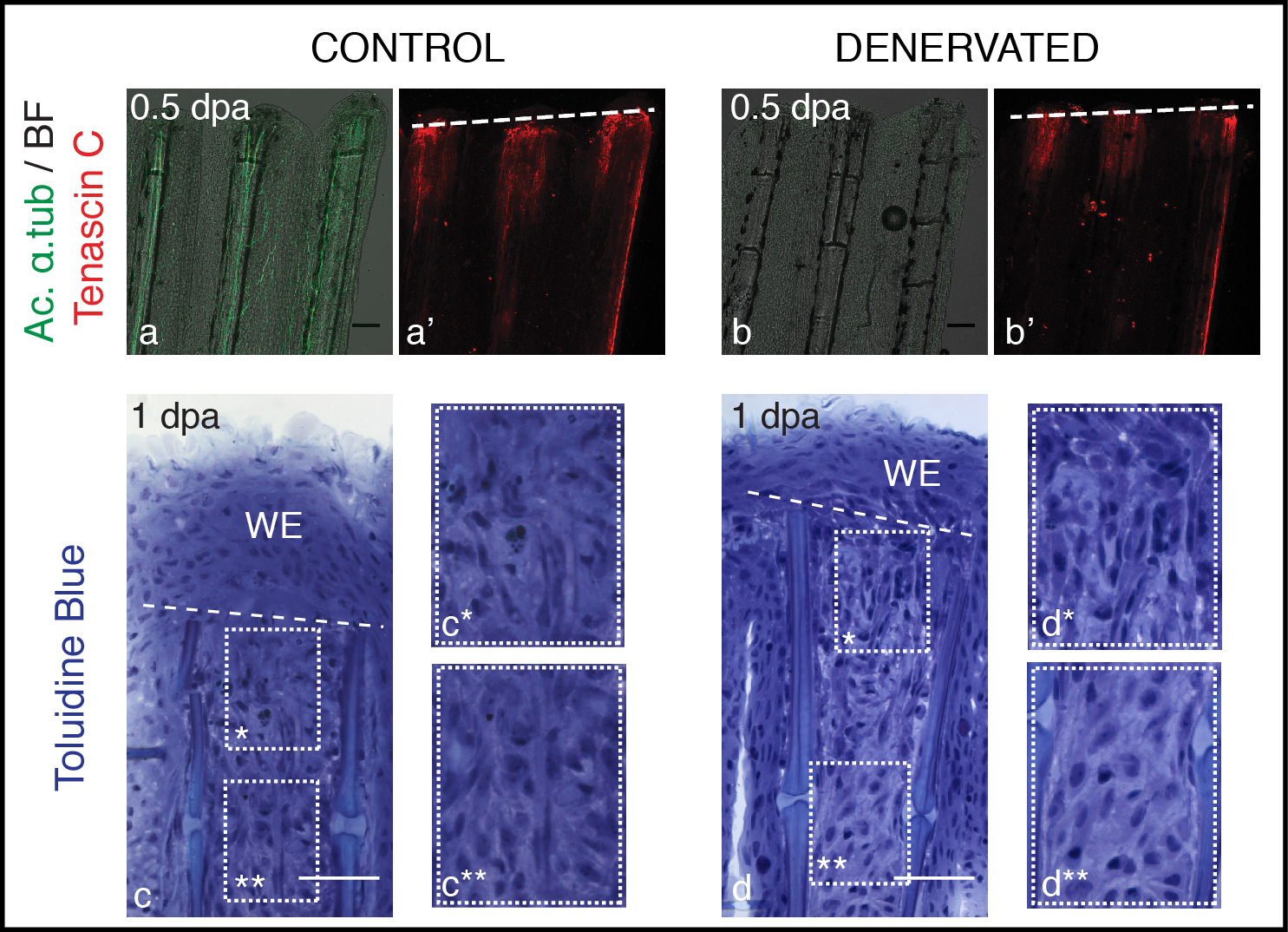

Supplement: Additional file 2: — Mesenchymal tissue disorganization after amputation of denervated fins. a,b) Staining for Tenascin C and ac. α-tub in whole mount fins at 0.5 dpa. Tenascin C is present in the mesenchymal tissue under the amputation plane, both in control and denervated fins. c,d) Toluidine Blue histology in longitudinal sections. Longitudinal sections stained with toluidine blue show that at 1 dpa mesenchymal tissue becomes more disorganised below the amputation plane, with cells presenting a more elongated shape that suggests cell migration, in both control (c*) and denervated fins (d*), while more proximal mesenchymal tissue presents a more organized structure (c**, d**). a-d) The images are a projection of confocal optical slices. Dashed lines mark amputation plane. a,b) Scale bar - 100 μm. c,d) Scale bar - 50 μm. [file 12861_2014_49_MOESM2_ESM.jpeg]

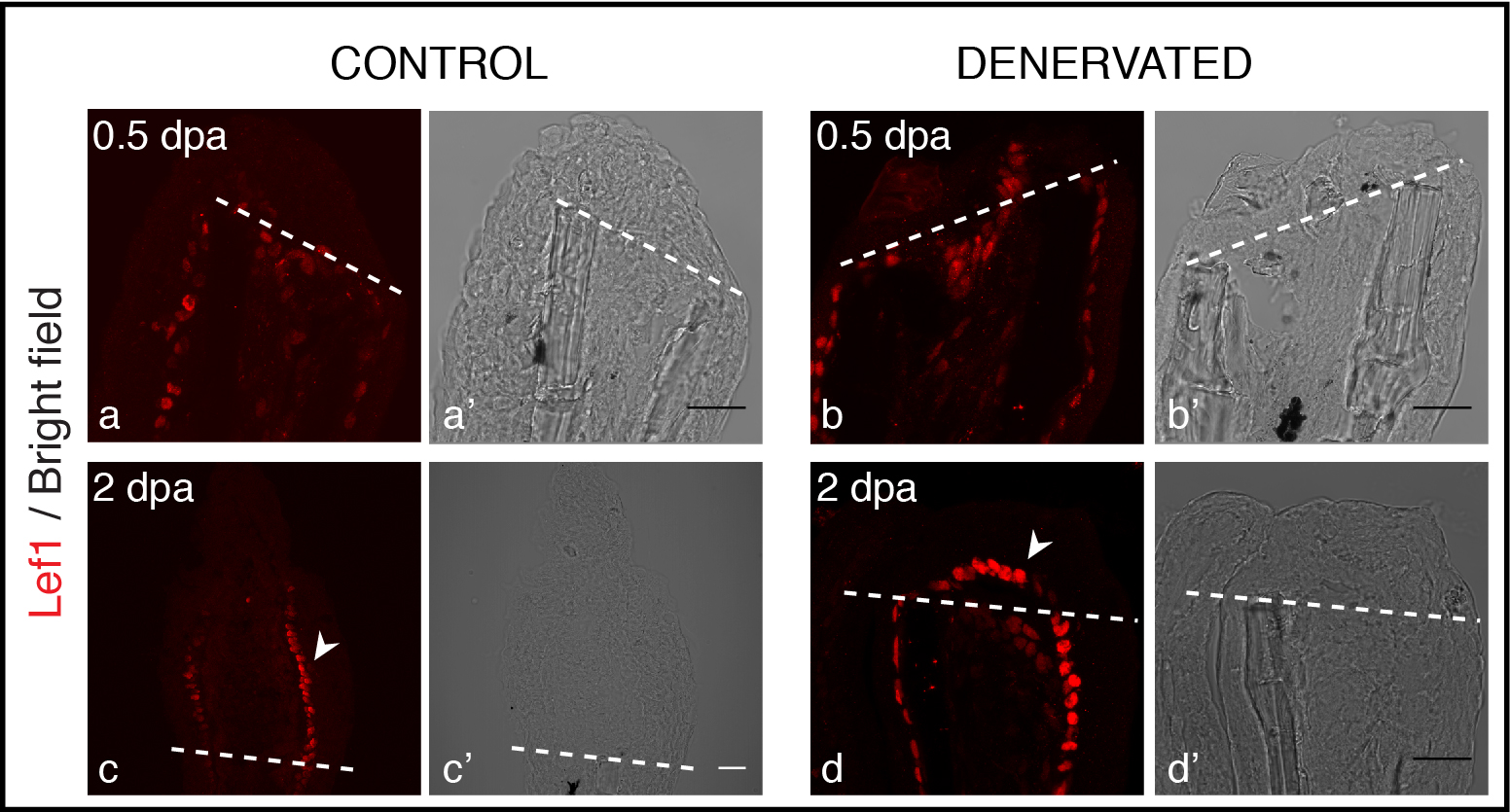

Supplement: Additional file 3: — Staining for Lef1 in longitudinal sections after amputation of denervated fins. Lef1 protein is detected in the BEL of both control and denervated fins from 0.5 to 2 dpa. At 0.5 dpa Lef1 expression is similar in control (a) and denervated (b) fins. At 2 dpa Lef1 is restricted to the most proximal BEL cells in control fins (c-arrowhead) and it is expressed all over the BEL lining mesenchymal cells of denervated fins (d-arrowhead). Scale bar - 25 μm. a-d) The images are a projection of confocal optical slices. Dashed lines mark amputation plane. Scale bar - 25 μm. [file 12861_2014_49_MOESM3_ESM.jpeg]

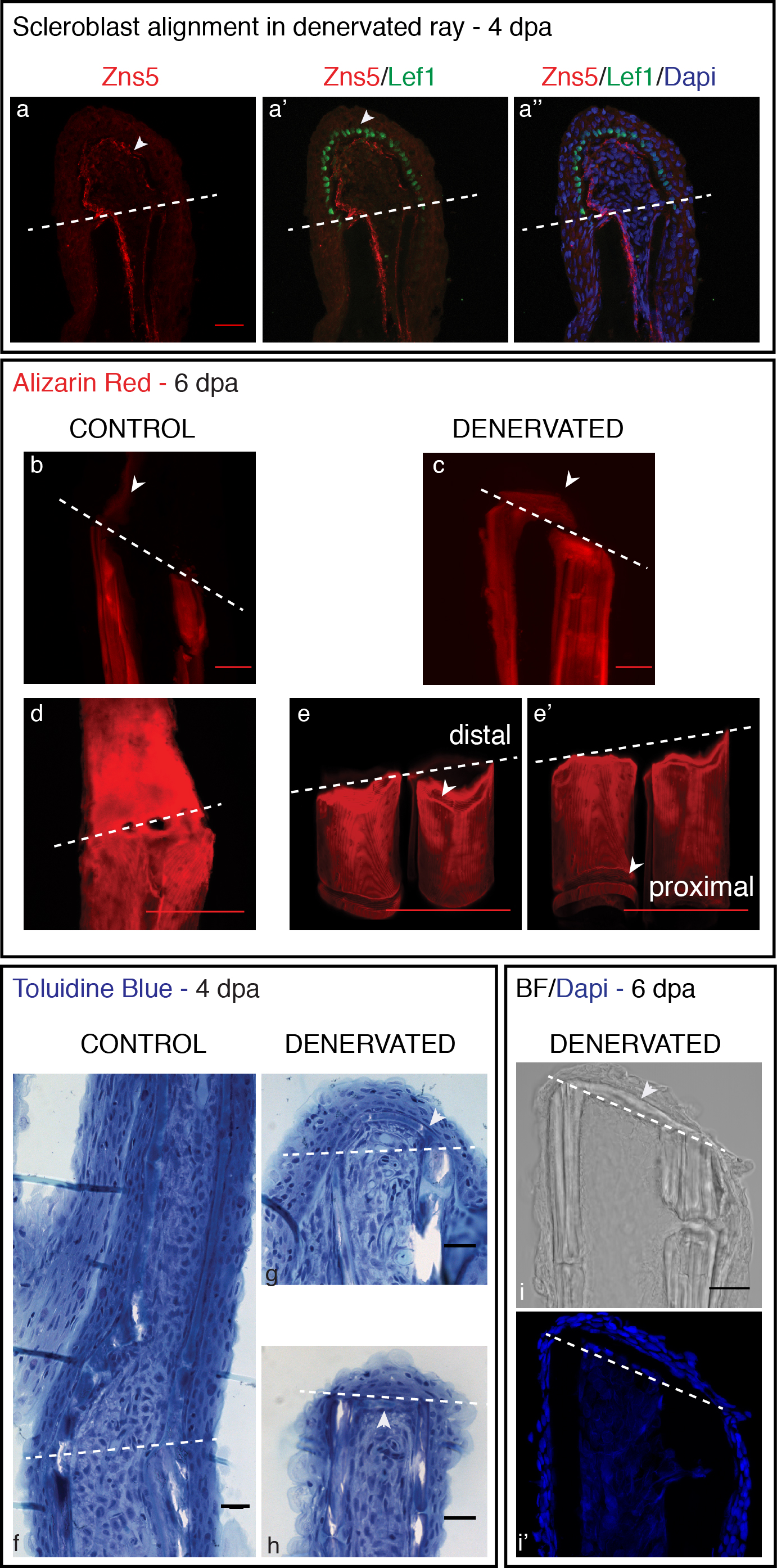

Supplement: Additional file 4: — Bone deposition in the amputation plane of denervated fins. a) Staining in longitudinal sections for Zns5, Lef1 and DAPI. Zns5-positive cells accumulate below the BEL of denervated fins, forming an arch that delimits mesenchymal cells. Lef1 is ectopically expressed in the whole BEL (a’). b,c) Staining with ARS in longitudinal sections. Alizarin Red staining at 6 dpa shows mineralization of the newly formed ray along the old bony ray in control fins (b-arrowhead), while in denervated fins it shows bone deposition in at the amputation level (c -arrowhead). d,e) Staining with ARS in whole mount fins. The deposition of bone cells in the amputation site causes a thickening in the distal stump of deneravated fins. A 3D projection of denervated rays shows that the distal region (e-arrowhead) is comparatively thicker in relation to the proximal region (e’-arrowhead). f-h) Toluidine Blue histology in longitudinal sections. At 4 dpa cell deposition between the 2 hemi-rays is observed in denervated fins (g,h). The new structure resembling bone matrix seems to be derived from the old bone (g - arrowhead) and a group of cells, resembling bone cells, are deposited at the level of amputation almost closing the ray (h - arrowhead). i) DAPI staining in longitudinal sections. At 6 dpa a bright field image shows matrix deposition at the level of amputation between the two hemi-rays (arrowhead). a-e, i) The images are a projection of confocal optical slices. Dashed lines mark amputation plane. a,e) Scale bar - 25 μm. b-d) Scale bar - 50 μm. [file 12861_2014_49_MOESM4_ESM.jpeg]

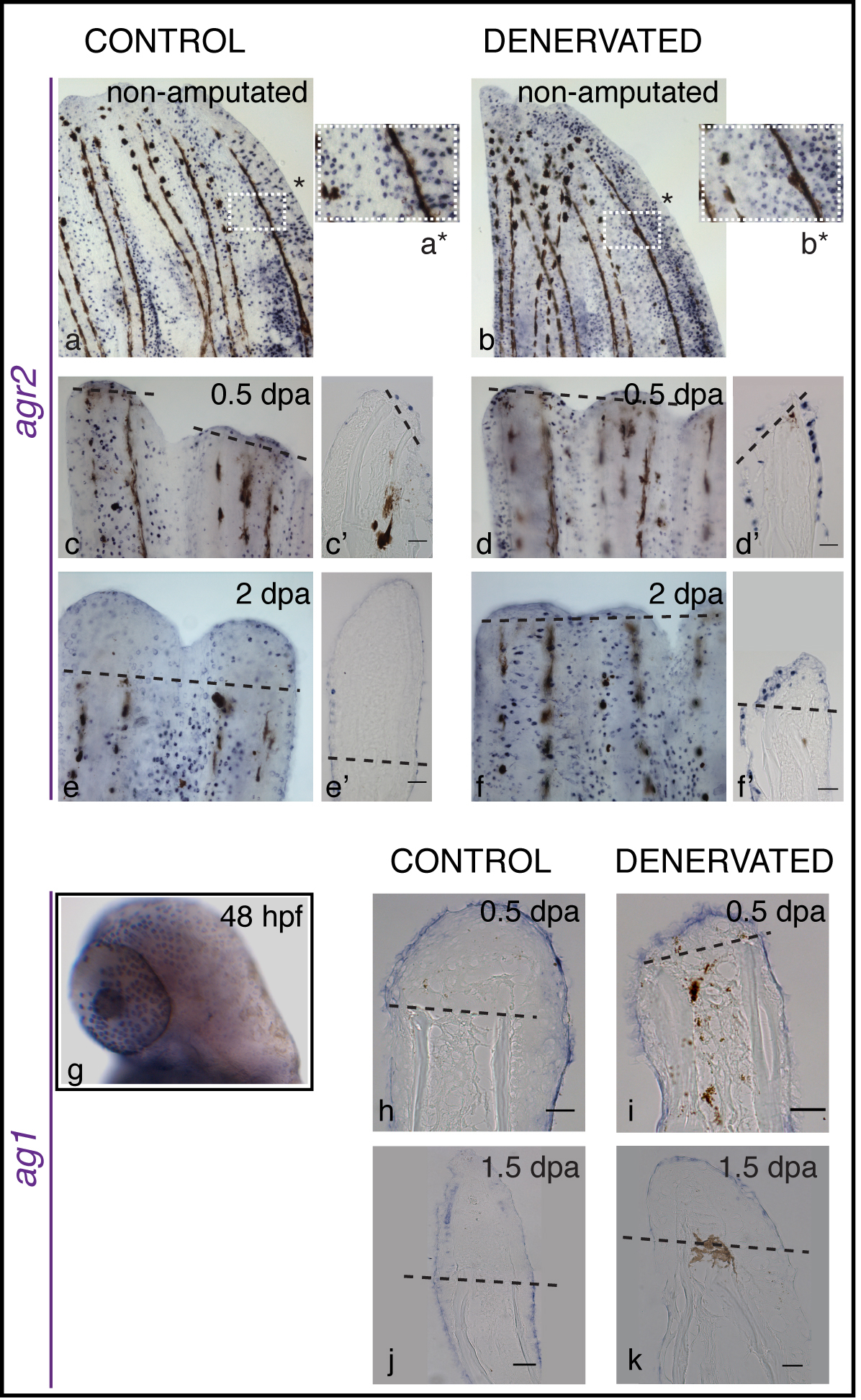

Supplement: Additional file 5: — Expression of nAg homologues in zebrafish. a-f) Whole mount mRNA ISH of agr2. c’-f’) Longitudinal sections of the rays using whole mount ISH. Agr2 mRNA is expressed in the mucous secreting cells of the entire epidermis in both non-amputated (a,b) and amputated fins (c-f). During regeneration, agr2 is also expressed in the mucous secreting cells present in the newly formed WE. There are no obvious differences between control (a,c,e) and denervated fins (b,d,f) in the amount of agr2- positive cells, in both non-amputated fins and during regeneration. g-k) Whole mount mRNA ISH of ag1. g) Ag1 mRNA is expressed in the mucous secreting cells of the larvae body at 48 hours post fertilization (hpf). h-k) Longitudinal sections of the rays using whole mount ISH show wide expression of ag1 in the epidermis and WE of adult fins, as early as 0.5 dpa, and throughout regeneration. There are no differences in ag1 expression between control and denervated fins, in both non-amputated and amputated fins. [file 12861_2014_49_MOESM5_ESM.jpeg]
